# *In silico* characterisation of lysis and host-recognition modules in *Staphylococcus aureus* bacteriophage genomes

**DOI:** 10.1590/0074-02760260245

**Published:** 2026-11-02

**Authors:** Ivan Armadi Hasugian, Na’ilah Insani Alifiyah

**Affiliations:** Universitas Terbuka, Faculty of Science and Technology, Department of Biology, Tangerang Selatan, Indonesia

**Keywords:** bacteriophages, Staphylococcus aureus, methicillin-resistant *Staphylococcus aureus*, genome, viral, phylogeny, host specificity

## Abstract

**BACKGROUND:**

Methicillin-resistant *Staphylococcus aureus* (MRSA) is a leading human pathogen, and declining antibiotic efficacy has renewed interest in lytic bacteriophages, whose efficacy phenotypic assays predict poorly.

**OBJECTIVES:**

To characterise the lysis and host-recognition modules of lytic *S. aureus* bacteriophages, and to determine how far the detectability of each module depends on the annotation source rather than on the genomes.

**METHODS:**

Twenty-two *S. aureus* phage genomes from NCBI GenBank were processed through an identical detection funnel under three annotation sources: the deposited record, Pharokka and Phold. Modules were validated by InterPro, disrupted endolysins resolved by tBLASTn, and each domain aligned separately to measure identity.

**FINDINGS:**

The LysK-type endolysin (CHAP-Amidase-SH3b) was conserved and detected identically under all three sources. Tail fibre/receptor-binding protein (RBP) detection was strongly source-dependent: 14 of 22 genomes from the deposited annotation, 20 of 22 after re-annotation, and 22 of 22 combined. Resolved by domain, conservation was scale-dependent: the amidase domain was invariant within species while SH3b varied by 5.7%.

**MAIN CONCLUSIONS:**

Lysis-module conservation is independent of the annotation source; the apparent variability of host recognition is largely an artefact of incomplete deposited annotation. Module-level surveys require re-annotation before comparative claims.

*Staphylococcus aureus*, and in particular methicillin-resistant *S. aureus* (MRSA), is among the most clinically important human bacterial pathogens, responsible for infections that range from skin and soft-tissue infections to life-threatening bacteraemia, endocarditis, and medical device-associated disease, and it is a high-priority target for the development of new antibacterial therapeutics.^(1)^ Antimicrobial resistance (AMR) driven by MRSA has escalated into a major global health threat, intensifying the need for precision non-antibiotic therapeutics.^(1^ ^,2)^ The structural resilience of MRSA, mediated by the mecA gene product PBP2a and by OatA-dependent peptidoglycan O-acetylation, undermines the efficacy of both conventional and last-line antibiotics.^(3^ ^,4)^ Lytic bacteriophages offer a rational alternative, delivering precise bactericidal activity through their lysis cassette — the holin and endolysin proteins that degrade peptidoglycan from within the host cell — without disrupting the commensal microbiota.^(5^ ^,6)^ Infection begins one step earlier, at the host-recognition cassette. The receptor-binding proteins (RBPs) of a tailed phage are the tail fibres, tailspikes and baseplate-associated proteins that make first contact with the bacterial surface; in *Staphylococcus* these recognise wall teichoic acid and its glycosylation pattern. RBPs are modular: an N-terminal region anchors the protein to the virion while a C-terminal region carries the receptor-binding specificity, so that the host range of a phage can be redirected by exchanging or mutating the C-terminal region alone, without altering the lytic enzymes.^(7^ ^,8)^ Lysis and host recognition are therefore complementary determinants of a phage’s therapeutic behaviour, and the present study characterises both.

Despite the conceptual elegance of phage lysis, phenotypic evaluation *in vitro* often gives unpredictable results, because several biological barriers intervene.^(9^ ^,10)^ A paradoxical observation illustrates this limitation: phage vB_SauP-436A markedly reduced MRSA biofilm biomass, whereas phage vB_SauM-515A1 increased biofilm biomass, even though both phages exhibited high planktonic lytic efficiency *in vitro*.^(11^ ^,12)^ Lytic efficacy alone is therefore an insufficient predictor of therapeutic success, particularly in chronic biofilm-associated infection.^(13)^

Complete genome sequences of *S. aureus* bacteriophages are now abundantly available in public repositories such as NCBI GenBank, and most tailed phages have recently been reclassified within the class Caudoviricetes, including the families Herelleviridae and Rountreeviridae, superseding the earlier morphology-based grouping.^(14)^ The product names in these deposited records, however, are often inconsistent with the underlying protein domain architecture. Re-examining the lysis and host-recognition modules against curated domain databases therefore offers an efficient and reproducible way to establish their functional content without isolating new phages.^(15^ ^,16)^

Although many *S. aureus* phage genomes are publicly available, the lysis and host-recognition modules of therapeutically relevant lytic phages have not been compared systematically. This study therefore set out to characterise the lysis and host-recognition modules of lytic *S. aureus* bacteriophages and to map candidate genomic features that may underlie inter-phage differences in therapeutic response. Because the dataset is limited to publicly deposited sequences, the study is descriptive and comparative. It is intended to provide a genomic foundation for selecting natural phage candidates and to inform later experimental work, not to dictate clinical formulation.

## MATERIALS AND METHODS

*Genome retrieval and dataset curation* - Genome sequences were retrieved from NCBI GenBank between 18 and 30 May 2026 using the search query ‘*Staphylococcus phage* complete genome’, supplemented by targeted retrieval of the specific strains examined in a previous study^(12)^ and downloaded as GenBank flat files. The initial set was curated against predefined criteria. The inclusion criteria were (i) records annotated as complete genome sequences, (ii) a lytic lifestyle inferred from genome annotation and the absence of lysogeny-associated genes (integrase, excisionase, CI repressor), and (iii) *S. aureus* as the annotated host. The exclusion criteria were (i) duplicate or near-identical genome sequences, (ii) the presence of lysogeny-associated genes, and (iii) jumbo genomes exceeding 200 kb. During curation, *Staphylococcus phage* vB_SauP_EBHT (NC_055906) was excluded because its intergenomic similarity to *Staphylococcus phage* Portland (MT926124) is 100.0%, and *Staphylococcus phage* MarsHill (MW248466) was excluded for exceeding 200 kb. The retained dataset was deliberately not dereplicated at the species level: because the object of study is the consistency of annotation rather than the extent of genomic diversity, genome pairs above the species demarcation threshold are informative rather than redundant, and their retention is what makes the annotation discordance reported below detectable. *Staphylococcus phage* Sb1M_6168 (MN336262) and Sb1M_9832 (MN336263) were retained on that basis as representatives of the Sb-1 lineage; the full similarity matrix is given in Supplementary data (Table I). One record, *Staphylococcus phage* vB_SauM-515A1 (MN047438), carried a Draft/Partial status flag and was retained as a single, explicitly noted exception to the complete-genome criterion because all modules analysed here are fully annotated and the phage is of direct comparative relevance to the previous study. Host identity of all 22 retained records was manually confirmed against the GenBank source entries prior to analysis. The final dataset comprised 22 genomes.

*Intergenomic similarity* - Nucleotide-level relatedness among the retained genomes, together with the two genomes excluded during curation, was quantified with VIRIDIC (Virus Intergenomic Distance Calculator)^(17)^ using the default BLASTN parameter set (-word_size 7 -reward 2 -penalty -3 -gapopen 5 -gapextend 2) and the demarcation thresholds recommended by the ICTV Bacterial and Archaeal Viruses Subcommittee, namely 95% intergenomic similarity for species and 70% for genus. Aligned genome fractions and genome length ratios were recorded alongside each similarity value, since a high similarity supported by a low aligned fraction does not indicate genome-wide relatedness.

*Genomic feature extraction* - Genome metadata were extracted using custom scripts written in Python 3.12.10 with Biopython 1.87^(18)^ and pandas 3.0.3. Using the Entrez and SeqIO modules, the scripts parsed each record to obtain genome size, GC content, the number of coding sequences (CDS), and the number of tRNA genes. Taxonomic ranks (class, family, subfamily) were resolved from the taxonomy field of each record by ICTV rank suffix (-viricetes, -viridae, -virinae); subfamilies placed by NCBI in no family (the Azeredovirinae phages EW and SA13) were recorded as family Unassigned. CDS counts are reported as deposited in GenBank; de novo re-annotation by independent gene callers may yield slightly different totals (for example, 236 versus 238 predicted features for MN047438). Transfer RNA counts in Table I are likewise those of the deposited record; they are reported separately from the de novo predictions described below, and the two are not interchangeable. Re-annotation and annotation-source comparison. All extracted values were verified manually against the source records and are summarised in Table I.

**TABLE I:** General characteristics of the 22 lytic *Staphylococcus aureus* bacteriophage genomes

Phage	Accession	Class	Family^*^	Subfamily	Genome size (bp)	GC (%)	CDS Total	tRNA count	NCBI status
*Staphylococcus phage* A5W	EU418428.2	Caudoviricetes	Herelleviridae	Twortvirinae	145,542	30.42	232	4	Complete
*Staphylococcus phage* qdsa002	KY779849.1	Caudoviricetes	Herelleviridae	Twortvirinae	142,499	30.26	231	0	Complete
*Staphylococcus phage* vB_SauM-fRuSau02	MF398190.1	Caudoviricetes	Herelleviridae	Twortvirinae	148,464	30.22	236	4	Complete
*Staphylococcus phage* vB_SavM_JYL01	MH159197.1	Caudoviricetes	Herelleviridae	Twortvirinae	141,384	30.17	219	0	Complete
*Staphylococcus phage* Maine	MN045228.1	Caudoviricetes	Herelleviridae	Twortvirinae	141,712	30.41	219	4	Complete
*Staphylococcus phage* vB_SauM-515A1	MN047438.1	Caudoviricetes	Herelleviridae	Twortvirinae	148,511	30.24	236	4	Draft/Partial
*Staphylococcus phage* vB_SauP-436A1	MN150710.1	Caudoviricetes	Rountreeviridae	Rakietenvirinae	18,028	29.34	21	0	Complete
*Staphylococcus phage* Sb1_8383	MN336261.1	Caudoviricetes	Herelleviridae	Twortvirinae	139,606	30.40	167	0	Complete
*Staphylococcus phage* Sb1M_6168	MN336262.1	Caudoviricetes	Herelleviridae	Twortvirinae	138,231	30.43	165	0	Complete
*Staphylococcus phage* Sb1M_9832	MN336263.1	Caudoviricetes	Herelleviridae	Twortvirinae	138,231	30.43	164	0	Complete
*Staphylococcus phage* Portland	MT926124.1	Caudoviricetes	Rountreeviridae	Rakietenvirinae	17,471	29.57	20	0	Complete
*Staphylococcus phage* Twort	NC_007021.1	Caudoviricetes	Herelleviridae	Twortvirinae	130,706	30.26	195	0	Complete
*Staphylococcus phage* EW	NC_007056.1	Caudoviricetes	Unassigned	Azeredovirinae	45,286	35.99	77	0	Complete
*Staphylococcus phage* JD007	NC_019726.1	Caudoviricetes	Herelleviridae	Twortvirinae	141,836	30.37	217	0	Complete
*Staphylococcus phage* SA13	NC_021863.1	Caudoviricetes	Unassigned	Azeredovirinae	42,652	35.42	62	0	Complete
*Staphylococcus aureus* bacteriophage Sb-1	NC_023009.1	Caudoviricetes	Herelleviridae	Twortvirinae	127,188	30.48	168	4	Complete
*Staphylococcus phage* Staph1N	NC_047722.1	Caudoviricetes	Herelleviridae	Twortvirinae	145,647	30.41	232	0	Complete
*Staphylococcus phage* A3R	NC_047723.1	Caudoviricetes	Herelleviridae	Twortvirinae	141,018	30.50	212	0	Complete
*Staphylococcus phage* 676Z	NC_047724.1	Caudoviricetes	Herelleviridae	Twortvirinae	148,564	30.41	233	0	Complete
*Staphylococcus phage* Fi200W	NC_047725.1	Caudoviricetes	Herelleviridae	Twortvirinae	148,481	30.39	233	0	Complete
*Staphylococcus phage* MSA6	NC_047726.1	Caudoviricetes	Herelleviridae	Twortvirinae	148,243	30.24	232	0	Complete
*Staphylococcus phage* P4W	NC_047727.1	Caudoviricetes	Herelleviridae	Twortvirinae	147,590	30.39	232	0	Complete

CDS counts are reported as deposited in GenBank; for MN047438 a de novo gene caller reports 238 features. *Family ‘Unassigned’ denotes a subfamily placed by NCBI/ICTV in no family (family incertae sedis), not a missing value.

*Functional annotation of lysis and host-recognition modules* - Endolysin-candidate proteins were extracted from each genome and submitted to InterPro Version 108.0^(19)^ for domain architecture analysis against the Pfam, CDD, SUPERFAMILY, and Gene3D databases; this identified the CHAP (cysteine, histidine-dependent amidohydrolase/peptidase), Amidase, SH3b (bacterial Src-homology 3b), LysM, and NlpC/P60 domains reported in Table II. Holin and tail fibre/receptor-binding protein (RBP) genes were detected from CDS product annotations using curated keyword sets, with each positive call retained together with the matched annotation for verification; the structural tail-tube protein, present in essentially all tailed phages, was deliberately excluded from the RBP keyword set. Because this procedure depends on the wording of the deposited product name, whereas endolysins were additionally validated at the domain level, the two module classes are not supported by evidence of equal strength. This asymmetry was addressed by re-annotation (see below), and RBP candidates were in addition submitted to InterPro under the same protocol used for endolysins, so that the evidence type supporting every call in Table II is stated explicitly. Where the automated candidate did not correspond to a recognised peptidoglycan hydrolase (Maine) or no intact endolysin-sized open reading frame was present (Sb1M_6168 and Sb1M_9832), the endolysin was treated as not confidently assigned by automated extraction; for the Sb-1 lineage the catalytic fragment was recovered by tBLASTn using the Sb1_8383 endolysin as query. In two further genomes, vB_SauM-515A1 (MN047438) and vB_SauM-fRuSau02 (MF398190), the endolysin (lysK) is annotated as one moiety because the gene is interrupted by a self-splicing intron; the intact enzyme is reconstituted at the RNA level, as demonstrated transcriptomically for vB_SauM-515A1,^(20)^ and the corresponding Table II entries are annotated accordingly. Targeted tBLASTn searches used the Sb1_8383 endolysin (MN336261, 495 aa) as query against each genome with BLAST+ 2.16.0, -outfmt 6 and -max_target_seqs 5, all other parameters at their defaults (e-value 10, word size 3, BLOSUM62, gap open 11, gap extend 1, SEG filtering on); a hit was accepted as the genuine endolysin at 90% identity or above. The outcome of each search is recorded in the host profile as a curated case with its identity value and literature source, so that re-running the pipeline reproduces the resolved assignment without repeating the search. Throughout, an endolysin is described as intact when its catalytic and cell-wall-binding domain architecture is complete in the mature protein, whether or not that protein is encoded by a single uninterrupted open reading frame; the term refers to domain architecture and not to sequence identity, which is reported separately in Supplementary data (Table II).

**TABLE II:** Domain architecture of lysis modules and presence of host-recognition modules among the 22 bacteriophages

Phage	Accession	Endolysin gene product	Endolysin length (aa)	Catalytic domains	Wall-binding domain	Holin present^e^	Tail fibre/RBP presente	RBP InterPro domain^f^
*Staphylococcus phage* A5W	EU418428.2	LysK	495	CHAP, Amidase	SH3b	Yes	Yes	No
*Staphylococcus phage* qdsa002	KY779849.1	Lysin	495	CHAP, Amidase	SH3b	Yes	No	-
*Staphylococcus phage* vB_SauM-fRuSau02	MF398190.1	LysK (intron-split)	209 (moiety; full LysK ~495)^a^	Amidase (moiety; CHAP intron-separated)	SH3b	Yes	Yes	Yes
*Staphylococcus phage* vB_SavM_JYL01	MH159197.1	Lysin	495	CHAP, Amidase	SH3b	Yes	Yes	Yes
*Staphylococcus phage* Maine	MN045228.1	LysK-type endolysin (recovered by tBLASTn; missed by keyword extraction)^c^	495 (tBLASTn vs Sb1_8383, 99% identity)	CHAP, Amidase	SH3b	Yes	Yes	Yes
*Staphylococcus phage* vB_SauM-515A1	MN047438.1	LysK (intron-split)	209 (moiety; full LysK ~495)^a^	Amidase (moiety; CHAP intron-separated)	SH3b	Yes	Yes	Yes
*Staphylococcus phage* vB_SauP-436A1	MN150710.1	Endolysin (CHAP-type)	250	CHAP	SH3b	No	Yes	Yes
*Staphylococcus phage* Sb1_8383	MN336261.1	Putative endolysin	494	CHAP, Amidase	SH3b	Yes	No	-
*Staphylococcus phage* Sb1M_6168	MN336262.1	Endolysin (HNH-disrupted)	141 (catalytic fragment, tBLASTn)^b^	CHAP	LysM (separately annotated)	Yes (putative)	No	-
*Staphylococcus phage* Sb1M_9832	MN336263.1	Endolysin (HNH-disrupted)	141 (catalytic fragment, tBLASTn)^b^	CHAP	LysM (separately annotated)	Yes (putative)	No	-
*Staphylococcus phage* Portland	MT926124.1	Lysin	249	CHAP	SH3b	Yes	Yes	Yes
*Staphylococcus phage* Twort	NC_007021.1	Tail-associated lysin (VAPH)^d^	1,269	Phage tail lysozyme (Phage_lysozyme2)	N/A (tail-anchored)	Yes	Yes	Yes
*Staphylococcus phage* EW	NC_007056.1	Endolysin (divergent)	482	Non-LysK (divergent)	-	Yes	Yes	Yes
*Staphylococcus phage* JD007	NC_019726.1	LysK (recovered by tBLASTn; missed by keyword extraction)^c^	495 (tBLASTn vs Sb1_8383, 99% identity)	CHAP, Amidase	SH3b	Yes	Yes	Yes
*Staphylococcus phage* SA13	NC_021863.1	Endolysin	481	CHAP, Amidase	SH3b	Yes	Yes	Yes
*Staphylococcus aureus* bacteriophage Sb-1	NC_023009.1	Endolysin	494	CHAP, Amidase	SH3b	Yes	Yes	Yes
*Staphylococcus phage* Staph1N	NC_047722.1	LysKn/LysKc	495	CHAP, Amidase	SH3b	Yes	Yes	No
*Staphylococcus phage* A3R	NC_047723.1	LysK	495	CHAP, Amidase	SH3b	Yes	Yes	No
*Staphylococcus phage* 676Z	NC_047724.1	LysK	495	CHAP, Amidase	SH3b	Yes	No	-
*Staphylococcus phage* Fi200W	NC_047725.1	LysK	495	CHAP, Amidase	SH3b	Yes	No	-
*Staphylococcus phage* MSA6	NC_047726.1	LysK	495	CHAP, Amidase	SH3b	Yes	No	-
*Staphylococcus phage* P4W	NC_047727.1	LysK	495	CHAP, Amidase	SH3b	Yes	No	-

*a*: for vB_SauM-fRuSau02 and vB_SauM-515A1 the *lysK* gene is interrupted by a self-splicing intron carrying the *ksaI* homing endonuclease; the 209-aa value is one moiety (Amidase + SH3b), and the intact LysK restores the CHAP–Amidase–SH3b architecture;^(17)^ *b*: for Sb1M_6168 and Sb1M_9832 the endolysin open reading frame is disrupted by an HNH endonuclease; the catalytic fragment was inferred by tBLASTn;^(22)^ *c*: for Maine and JD007, automated extraction returned a non-lytic YbiA-like N-glycosidase and a virion-associated NlpC/P60 protein, respectively; the genuine LysK endolysins (495 aa, 99% identity to Sb1_8383) were recovered by tBLASTn; *d*: for Twort, tBLASTn detected only a partial, divergent LysK region (45% identity over 456 aa), and its principal hydrolase is the virion-associated tail lysozyme; *e*: holin and RBP presence were called from the wording of the deposited CDS product annotation, not from domain assignment (see Table III for how both calls change under re-annotation); *f*: RBP candidates from the deposited annotation submitted to InterPro under the same protocol as endolysins; Yes: at least one IPR entry returned; No: candidate submitted but no IPR entry; dash: no RBP candidate to submit. Domain support was obtained for 11 of 22 genomes; *g*: dndolysin domain architecture was validated by InterPro; holin and RBP presence was called from CDS product annotation, with InterPro domain support indicated per entry where obtained; VAPH: virion-associated peptidoglycan hydrolase; RBP: receptor-binding protein.

*Domain-partitioned pairwise identity* - To quantify what “conserved” refers to, the unique endolysin sequences were partitioned at their InterPro-resolved domain boundaries and each domain set was aligned separately, so that variation in one domain could not be averaged away by another. Boundaries were taken from the InterPro output already generated for Table II rather than re-predicted; where several member databases annotated the same domain, the widest span was used and its source recorded (CHAP, PS50911; amidase, G3DSA:3.40.80.10; SH3b, G3DSA:2.30.30.40, the Pfam amidase signature PF01510 being returned for too few sequences to define the set). Each partition, and the full-length sequences, were aligned on the MAFFT online service with the L-INS-i strategy^(20)^ and pairwise percent identity was computed over columns in which both sequences carry a residue, columns with a gap in either sequence being excluded from numerator and denominator alike. Because the three domains were not resolved in the same set of proteins, identity was compared across domains only within the subset carrying all three.

*Phylogenetic analysis* - Phylogenetic relationships were inferred from the large terminase subunit (TerL), a conserved marker suitable for resolving relationships across phage families.^(21^ ^,22)^ TerL sequences were extracted from each genome; an exact-match check was required to capture the abbreviated ‘Ter’ product name used in the Kayvirus group. Where a genome carried more than one terminase-annotated coding sequence, the longest was taken, since terminase genes are frequently split across several open reading frames and returning the first match recovers a fragment while the full-length product lies further along the genome. This affected Twort (NC_007021), whose terminase is interrupted by HNH endonuclease insertions and annotated as three separate coding sequences; the longest fragment (286 aa against a 605-aa reference) was used, InterPro returned no domain for it, and the position of Twort in Figure therefore rests on partial data. Its separation from the remaining genomes is independently supported by intergenomic similarity (34.5-36.8%, below the ICTV genus demarcation threshold). *Staphylococcus phage* Portland (MT926124) and vB_SauP-436A1 (MN150710) lacked an annotated TerL — consistent with their atypical small genome sizes (< 20 kb) — and were excluded, leaving 20 sequences. Sequences were aligned on the MAFFT online service using the L-INS-i strategy with all other settings at their defaults.^(20)^ A maximum-likelihood tree was constructed in MEGA 12.1.2^(23)^ under the LG+G+I substitution model, with rate variation modelled by a discrete Gamma distribution and a proportion of invariant sites; node support was assessed with 1000 bootstrap replicates, gaps were treated by partial deletion with an 80% site-coverage cutoff, and the tree was rooted on the Azeredovirinae lineage, represented here by *Staphylococcus phage*s EW (NC_007056) and SA13 (NC_021863), which are recovered as a pair at 100% bootstrap and are the only genomes in the dataset placed by NCBI in no family. The tree was annotated in iTOL with five tip-level layers drawn from Tables I, II and III: family, genome size, endolysin domain architecture, lysis-gene disruption (HNH insertion and intron split), and the receptor-binding protein call before and after re-annotation.

**TABLE III:** Detection of holin and receptor-binding protein (RBP) across three annotation sources in 22 lytic *Staphylococcus aureus* bacteriophage genomes

Phage	Accession	Size (kb)	Holin GB	Holin Phk	Holin Phd	RBP GB	RBP Phk	RBP Phd	RBP loci GB/Phk/Phd
676Z	NC_047724.1	148.6	Yes	Yes	Yes	No	Yes	Yes	0 / 3 / 3
vB_SauM-515A1	MN047438.1	148.5	Yes	Yes	Yes	Yes	Yes	Yes	2 / 3 / 3
Fi200W	NC_047725.1	148.5	Yes	Yes	Yes	No	Yes	Yes	0 / 3 / 3
vB_SauM-fRuSau02	MF398190.1	148.5	Yes	Yes	Yes	Yes	Yes	Yes	2 / 3 / 3
MSA6	NC_047726.1	148.2	Yes	Yes	Yes	No	Yes	Yes	0 / 3 / 3
P4W	NC_047727.1	147.6	Yes	Yes	Yes	No	Yes	Yes	0 / 3 / 3
Staph1N	NC_047722.1	145.6	Yes	Yes	Yes	Yes	Yes	Yes	1 / 3 / 3
A5W	EU418428.2	145.5	Yes	Yes	Yes	Yes	Yes	Yes	1 / 3 / 3
qdsa002	KY779849.1	142.5	Yes	Yes	Yes	No	Yes	Yes	0 / 3 / 3
JD007	NC_019726.1	141.8	Yes	Yes	Yes	Yes	Yes	Yes	3 / 3 / 3
Maine	MN045228.1	141.7	Yes	Yes	Yes	Yes	Yes	Yes	1 / 3 / 3
VB_SavM_JYL01	MH159197.1	141.4	Yes	Yes	Yes	Yes	Yes	Yes	1 / 3 / 3
A3R	NC_047723.1	141.0	Yes	Yes	Yes	Yes	Yes	Yes	1 / 3 / 3
Sb1_8383	MN336261.1	139.6	Yes	Yes	Yes	No	Yes	Yes	0 / 3 / 3
Sb1M_6168	MN336262.1	138.2	Yes	Yes	Yes	No	Yes	Yes	0 / 3 / 3
Sb1M_9832	MN336263.1	138.2	Yes	Yes	Yes	No	Yes	Yes	0 / 3 / 3
Twort	NC_007021.1	130.7	Yes	Yes	Yes	Yes	Yes	Yes	3 / 3 / 3
Sb-1	NC_023009.1	127.2	Yes	Yes	Yes	Yes	Yes	Yes	3 / 3 / 3
EW	NC_007056.1	45.3	Yes	Yes	Yes	Yes	Yes	Yes	1 / 1 / 2
SA13	NC_021863.1	42.7	Yes	Yes	Yes	Yes	Yes	Yes	2 / 2 / 2
vB_SauP-436A1	MN150710.1	18.0	No	Yes	Yes	Yes	No	No	2 / 0 / 0
Portland	MT926124.1	17.5	Yes	Yes	Yes	Yes	No	No	3 / 0 / 0
Total			21/22	22/22	22/22	14/22	20/22	20/22	26 / 57 / 58

GB: annotation as deposited in GenBank; Phk: Pharokka v1.10.1; Phd: Phold v1.3.0. All three arms were processed through the identical detection funnel; only the source of CDS product names differed. Union: detected in at least one of the three sources; Holin: 22/22; RBP: 22/22. No genome was negative in all three. Endolysin detection was identical in all three arms: an endolysin representative could be selected from the annotation in the same 18 genomes in every arm, collapsing to the same 11 unique sequences. In the remaining four genomes the endolysin is present in the genome but is not represented by a single annotated coding sequence, either because it was recovered by tBLASTn (MN045228, NC_019726) or because the open reading frame is interrupted by an HNH endonuclease insertion (MN336262, MN336263) (see Table II footnotes b and c). Endolysin detection is therefore not repeated here. InterPro domain support for RBP candidates was obtained for 11/22 genomes in each arm, although not for the same 11 genomes: eight were supported in all three arms and the union across arms was 14/22. vB_SauP-436A1 and Portland are RBP-positive in GB and negative after re-annotation (see Results for the explanation).

**Fig.:**
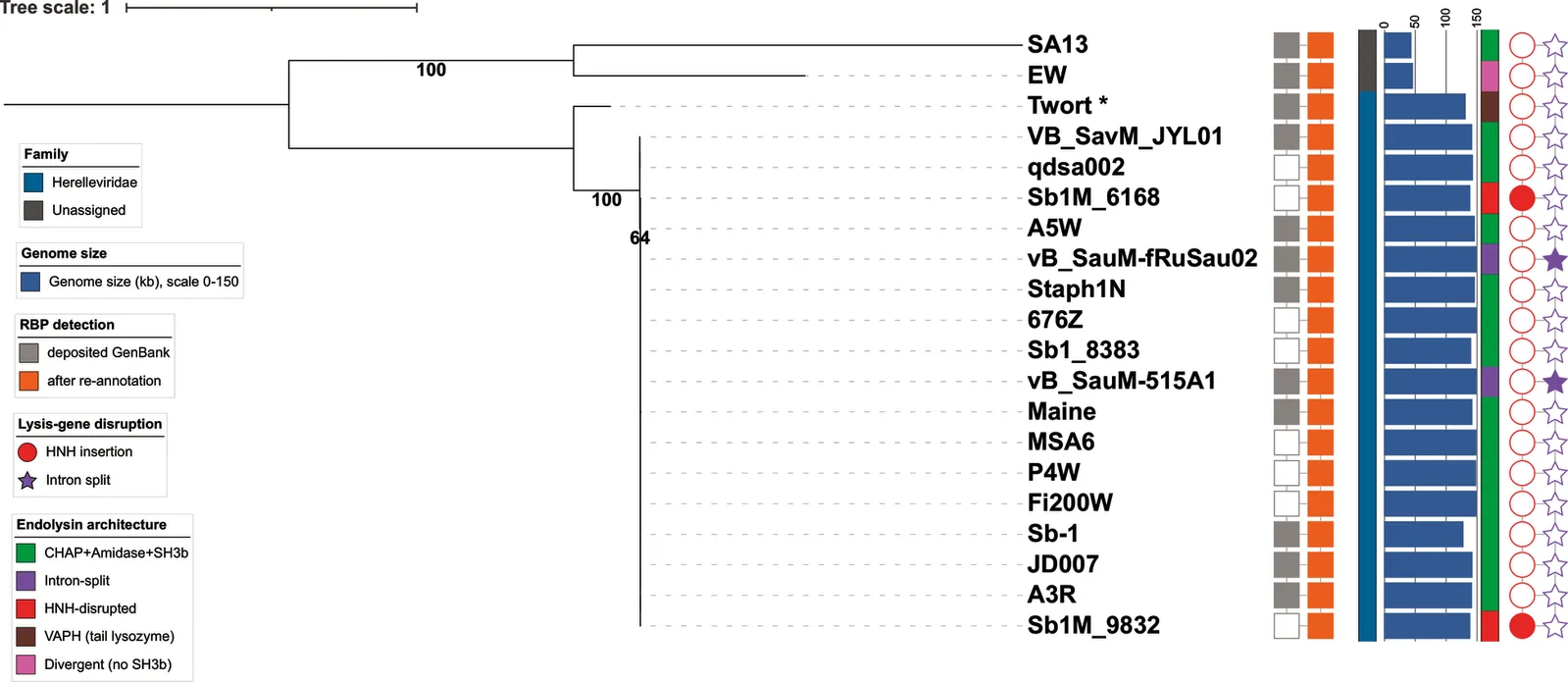
Maximum-likelihood phylogenetic tree of 20 Staphylococcus aureus bacteriophages based on large terminase subunit (TerL) sequences, inferred in MEGA 12.1.2 under the LG+G+I model with 1000 bootstrap replicates, rooted on the Azeredovirinae lineage (EW, NC_007056 and SA13, NC_021863) and annotated in iTOL. Tip labels give the phage name; accessions are listed in Table I. Tip annotations, from left to right: the receptor-binding protein call, where the grey square is the call from the deposited annotation and the orange square the call after re-annotation; family; genome size in kb; endolysin domain architecture; and lysis-gene disruption, where a filled circle marks an HNH endonuclease insertion and a filled star an intron-split lysK. The eight genomes carrying an empty grey square beside a filled orange one are those whose receptor-binding protein was recovered only after re-annotation, and they fall within the group whose members are at or above the species demarcation threshold to one another. Bootstrap values are shown at the nodes: support is 100% for the 17-phage group and for the EW-SA13 pair, and 27-64% for nodes within the 17-phage group, where internal branch lengths approach zero. An asterisk marks Twort, represented by the longest of the three coding sequences into which its terminase is split (286 aa). Portland (MT926124) and vB_SauP-436A1 (MN150710) carry no terminase-annotated coding sequence and are not included. Scale bar, substitutions per site.

*Re-annotation and annotation-source comparison* - To test whether module detection reflects genomic content or annotation completeness, all 22 genomes were re-annotated independently of their deposited records and re-processed through the identical detection funnel, so that the annotation layer was the only variable. Structural gene calling and functional assignment were performed with Pharokka v1.10.1;^(24)^ the resulting GenBank records were passed to Phold v1.3.0,^(25)^ which assigns function by ProstT5 protein-language-model embeddings followed by Foldseek structural search against the Phold database, executed on an NVIDIA RTX 4060 GPU at half precision (PyTorch 2.8.0+cu128); residual proteins still annotated as hypothetical after Phold were passed to Phynteny_transformer v0.1.3, which assigns PHROG (Prokaryotic Virus Remote Homologous Groups) functional categories from genomic context using ESM-2 embeddings. Phynteny returns a PHROG category rather than a product name and was therefore not used to make module calls; its output is reported separately in Supplementary data (Table III). Three detection arms were compared: the deposited GenBank annotation, the Pharokka re-annotation, and the Phold re-annotation. All three used the same keyword sets, the same InterPro protocol and the same curation funnel; only the source of the CDS product names differed. Results are given in Table III.

*Codon usage and phage-encoded tRNAs* - Relative synonymous codon usage (RSCU)^(26)^ was computed for the host by pooling the coding sequences of five curated *S. aureus* reference genomes (NCTC 8325, NC_007795.1; Mu50, NC_002758.2; MRSA252, NC_002952.2; USA300_FPR3757, NC_007793.1; and CP023390.1) rather than a single strain, and, separately, for each phage genome from its complete CDS complement, using a single shared implementation so that the definition could not differ between host and phage. Similarity between each phage and the host was quantified over the 59 codons belonging to degenerate synonymous families (methionine and tryptophan excluded, being invariant at RSCU = 1) as the Pearson correlation and the Spearman rank correlation of the two RSCU vectors, together with GC content at the third codon position (GC3) and the mean absolute RSCU difference. Transfer RNA genes were predicted de novo with tRNAscan-SE 2.0^(27)^ rather than read from the deposited annotation, and each predicted anticodon was mapped to the codons it decodes and to the host RSCU of those codons; a codon was classified as rare in the host at RSCU < 0.5.

*Ethics* - This study is entirely computational and analysed only publicly available genome sequences; no ethics approval was required and no biological material was accessed.

## RESULTS

*General genomic characteristics of lytic S. aureus* bacteriophages - The 22 complete bacteriophage genomes retrieved from NCBI GenBank varied substantially (Table I). Genome size ranged more than eight-fold, from 17,471 bp in *Staphylococcus phage* Portland (MT926124) to 148,564 bp in *Staphylococcus phage* 676Z (NC_047724). GC content varied within a narrower range, from 29.34% in vB_SauP-436A1 (MN150710) to 35.99% in phage EW (NC_007056), and the number of predicted CDS ranged from 20 in phage Portland to 236 in both vB_SauM-fRuSau02 (MF398190) and vB_SauM-515A1 (MN047438). On the basis of genome size and CDS count, the phages fell into three size classes: a micro class (17-18 kb, 20-21 CDS) comprising Portland and vB_SauP-436A1, both assigned to the family Rountreeviridae; a medium class (42-45 kb, 62-77 CDS) comprising phages EW and SA13; and a macro class (127-148 kb, 164-236 CDS) encompassing the majority of the dataset, including the Kayvirus group and the Sb-1 lineage. GC content was tightly constrained: 20 of the 22 phages fell below 31%, slightly below the 32-33% genomic GC of *S. aureus*. Two phages, EW (35.99%) and SA13 (35.42%), were clear outliers with GC content above the host average. Transfer RNA genes were annotated in only five of the deposited records (four tRNAs each in vB_SauM-fRuSau02, A5W, vB_SauM-515A1, Sb-1 and Maine), whereas de novo scanning recovered tRNA genes in 18 of the 22 genomes: three tRNAs (Asp-GTC, Ile2-CAT, Phe-GAA) in 17 macro-class genomes and a single Ile2-CAT in Twort. The four genomes returning no tRNA de novo were the two micro-class genomes, Portland and vB_SauP-436A1, and the two medium-class genomes, EW and SA13. Per-genome counts from the de novo scan are provisional: the calibration control recovered three of its four expected tRNAs, so the absolute number per genome may be underestimated by one, although the presence/absence pattern across genomes is unaffected.

*Lysis modules and the source-dependence of host-recognition detection* - Functional annotation of the 22 genomes revealed high conservation of the intracellular lysis components, and an apparent variability of the extracellular host-recognition components that the following section shows to be a property of the deposited annotation rather than of the genomes (Table II). Among phages with a canonical free endolysin, protein length clustered tightly around 480-495 amino acids, and fourteen of these displayed the classic LysK-type architecture, defined by tandem N-terminal catalytic domains (CHAP and Amidase) and a C-terminal SH3b cell wall-binding domain (CBD); proteins falling outside this range corresponded to specific structural or genomic exceptions rather than to true size variation of the endolysin. In the deposited annotation, holin was detected in 21 of the 22 phages and tail fibre or RBP genes in 14 of the 22. As shown in the following section, both figures are properties of the deposited annotation rather than of the genomes, and neither should be read as a measure of biological variability. The conserved LysK-type architecture extended even to the phylogenetically divergent phage SA13 (481 amino acids), which retained the full CHAP-Amidase-SH3b organisation despite its long branch and elevated GC content; the principal exception was phage EW, the most divergent lineage, whose 482-amino-acid endolysin diverges from the canonical LysK architecture.

Two mechanistically distinct departures from the canonical architecture were observed, both of which confound keyword-based annotation. In the Sb-1 lineage (Sb1M_6168, Sb1M_9832) the endolysin open reading frame is interrupted by an HNH endonuclease insertion, leaving no intact endolysin-sized frame in the deposited inventory; in vB_SauM-515A1 and vB_SauM-fRuSau02 the lysK gene is interrupted by a self-splicing group I intron carrying the ksaI homing endonuclease, and the intact CHAP-Amidase-SH3b enzyme is reconstituted at the RNA level.^(28^ ^,29)^ In three further genomes the keyword extractor returned a protein that was not the free endolysin - a non-lytic YbiA-like N-glycosidase in Maine, and virion-associated peptidoglycan hydrolases in JD007 and Twort - and a targeted tBLASTn search using the Sb1_8383 endolysin as query was required to resolve each case, recovering a canonical 495-amino-acid LysK at 99% identity in Maine and JD007 but only a partial, divergent region in Twort.^(30)^ The individual cases are described in Supplementary data.

Conservation is reported here as pairwise amino-acid identity within a defined domain, not as the mere presence of a domain, since a domain can be present in every protein while its sequence varies widely. Partitioning the endolysins on that basis shows that “conserved” applies at two different scales and with opposite rank order. Six of the unique sequences carried all three domains and could be compared directly. Among the five drawn from macro-class genomes, which are themselves at or above the species demarcation threshold [Supplementary data (Table I)], the amidase domain was invariant at 100% identity across all ten pairs, CHAP ranged from 99.2 to 100%, and SH3b from 94.3 to 100%, making SH3b the only domain to fall below 95% at this scale. Between SA13 and those five sequences the order reverses: CHAP is the most divergent domain at 22.9% identity in all five pairs, SH3b intermediate at 47.6-48.6%, and the amidase domain the least divergent at 54.2-54.5%, against 42.7-43.0% for the full-length proteins. The distribution is strictly bimodal, with ten pairs above 94% and five below 60% and none between, so a single summary statistic across all pairs would describe neither population [Supplementary data (Table II)].

*Detection of lysis and host-recognition modules depends on the annotation source* - Re-processing the same 22 genomes through the identical funnel under three annotation sources changed the detection of both host-recognition and holin genes, while leaving endolysin detection unaffected (Table III). Holin rose from 21 of 22 in the deposited annotation to 22 of 22 under both Pharokka and Phold. Tail fibre/RBP genes rose from 14 of 22 to 20 of 22 under both re-annotations, and the total number of RBP loci rose from 26 to 57 (Pharokka) and 58 (Phold). Taking the union of the three sources, holin and RBP were each detected in 22 of 22 genomes; no genome was negative in all three. The change is concentrated in the Sb-1 lineage and the Kayvirus group. Sb1_8383, Sb1M_6168 and Sb1M_9832 each moved from no detected RBP locus to three, while Sb-1 itself remained at three, removing a discordance that had appeared within a single lineage. Five further macro-class genomes (676Z, Fi200W, MSA6, P4W, qdsa002) moved from zero to three loci.

*Two genomes moved in the opposite direction* - vB_SauP-436A1 and Portland, the two micro-class Rountreeviridae genomes, were RBP-positive in the deposited annotation but negative under both re-annotations. Inspection of the underlying calls indicates that the product names assigned by Pharokka and Phold to the short tail fibres of these genomes fall outside our keyword set; this is a limitation of the detection layer applied here, not a biological difference, and it is reported as such. Both genomes are positive in the union column. Endolysin detection was invariant across the three sources: an endolysin representative was selected in the same 18 genomes in every arm, collapsing to the same 11 unique sequences and the domain architectures reported in Table II were unchanged. InterPro domain support was recovered for the RBP candidates of 11 of 22 genomes in each arm, although not for the same 11: eight genomes were supported in all three arms, and the union across arms was 14 of 22. In every arm the domain-supported figure is lower than the keyword-based call, consistent with the sparse representation of receptor-binding protein folds in profile-HMM databases and with structural homology being the more appropriate comparator for this module class. Passing the Phold records through Phynteny and re-processing the resulting 22 genomes through the same funnel changed nothing: holin was detected in 22 of 22 genomes, tail fibre/RBP in 20 of 22, and the number of RBP loci was 58, matching the Phold arm genome for genome in all three measures. Phynteny is therefore reported as the residual annotation it resolves [Supplementary data (Table III)] rather than as a fourth detection arm.

*Intergenomic similarity provides an annotation-independent test of this interpretation* - Across the 22 retained genomes, 67 of the 231 genome pairs reach or exceed the ICTV species demarcation threshold of 95%, and three pairs reach 100.0%: A5W with Staph1N, Sb1M_6168 with Sb1M_9832, and the excluded Portland with vB_SauP_EBHT. Of those 67 species-level pairs, 38 are discordant in the deposited RBP call, one genome being scored positive and the other negative, the most extreme case being vB_SauM-fRuSau02 and MSA6 at 99.8% intergenomic similarity. Genome pairs that are 99-100% identical at the nucleotide level cannot differ in the presence or absence of an entire gene family, so these discordances are necessarily artefacts of the deposited annotation rather than genuine differences in gene content. Because this test uses no re-annotation of any kind, it corroborates Table III independently.

At the genus demarcation threshold of 70%, the dataset resolves into six clusters: a single macro-class cluster of 17 genomes; a Rountreeviridae cluster containing vB_SauP-436A1, Portland and the excluded vB_SauP_EBHT; and Twort, EW and SA13 each alone. Twort shares only 34.5-36.8% intergenomic similarity with the macro-class cluster despite its basal position within the TerL core clade, and EW and SA13 share 27.4% with each other and no more than 0.5% with any other genome, consistent with their long branches and elevated GC content [Supplementary data (Table I)].

*Phylogenetic relationships based on the large terminase subunit* - The maximum-likelihood tree inferred from TerL sequences, rooted on phage EW, separated the 20 analysed phages into a supported core group, a basal lineage and a divergent pair (Figure). Seventeen phages with large genomes (127-148 kb) consistent with a myovirus-type morphology formed a group supported at 100% bootstrap, within which internal branch lengths were near zero and internal node support ranged from 27 to 64%. *Staphylococcus phage* Twort (NC_007021) fell outside that group as its immediate sister lineage, consistent with its recognised status as a prototype myovirus and with its intergenomic similarity to the group (34.5-36.8%) lying below the genus demarcation threshold. Phages EW (NC_007056) and SA13 (NC_021863) formed the divergent pair, joined at 100% bootstrap, with terminal branches of 0.797 and 1.545 substitutions per site respectively and a shared internal branch of 1.961. Of the 190 pairwise TerL distances, 92 were exactly zero. The tree therefore resolves lineage separation but not branching order within the 17-phage group, and Figure should be read accordingly.

*Codon usage similarity to the host is stratified by size class and coincides with tRNA carriage* - Codon usage of every phage was compared with that of *S. aureus* [Supplementary data (Table IV)]. Similarity was high overall but not uniform, the Pearson correlation of RSCU vectors ranging from 0.844 to 0.972 (Spearman 0.797-0.956). The dataset separated completely into two groups with no intermediate value. The four genomes carrying no de novo-detectable tRNA — vB_SauP-436A1, Portland, EW and SA13 — showed the closest correspondence to host codon usage (r = 0.927-0.972). The 18 genomes carrying tRNAs showed the lowest (r = 0.844-0.881), the two ranges being separated by a gap between 0.881 and 0.927.

This ordering is not reducible to base composition. Host GC3 was 22.73%; the two micro-class genomes lay closest to it (21.28% and 21.31%), but EW and SA13, whose GC3 of 30.62% and 29.85% departs from the host by more than seven percentage points, nonetheless resembled the host more closely in RSCU than the macro-class genomes, whose GC3 of 18.70-20.37% lies within four points of the host value. Codon preference within synonymous families therefore carries information that GC3 alone does not. The largest single contributions to the macro-class divergence were an excess of AGA against a deficit of CGT among the arginine codons, and an excess of CCT against a deficit of CCA among the proline codons.

The tRNA complement of the macro-class genomes was uniform: Asp-GTC, Ile2-CAT and Phe-GAA, decoding five codons, of which one (GAC) is rare in the host at RSCU < 0.5. In every case the remaining members of the same synonymous family were also decoded, so the complement does not constitute selective supplementation of rare host codons; it is equally consistent with generic translational support and with the proposed anti-defence role, and the present sequence-based analysis does not distinguish between them.

## DISCUSSION

Genomic characterisation stratifies these phages in a way that phenotypic data alone cannot. The earlier experimental evaluation of lytic activity^(12)^ did not treat genomic capacity as a variable influencing therapeutic outcome. Yet the more than eleven-fold difference in CDS count between Portland (20 CDS) and vB_SauM-fRuSau02 (236 CDS) implies large differences in coding capacity for lysis strategy, accessory functions and engineering potential, even though the accessory-gene repertoires behind those differences were not annotated here. This stratification is consistent with established morphological descriptions, in which larger contractile-tailed (myovirus-type) phages correspond to the macro class and smaller phages to the micro class; macro-class phages possess sufficient coding capacity to accommodate additional engineering modules, an advantage absent in micro-class phages whose genomes approach the functional minimum.^(31)^

The conserved LysK-type architecture acts cooperatively to cleave the pentaglycine cross-bridges characteristic of the *S. aureus* peptidoglycan,^(32)^ and its conservation even in the divergent phage SA13 underscores the strong selective constraint on the lytic enzyme. Against this conserved background, the two independent mechanisms of gene interruption identified here — HNH endonuclease insertion in the Sb-1 lineage and self-splicing intron interruption of lysK in the Kayvirus group — are notable chiefly because they mislead annotation that relies on product names alone: in both cases the intact catalytic architecture is preserved, and the apparent anomaly is an artefact of the deposited annotation rather than a functional deficiency. Reliable module assignment therefore requires domain-level validation and homology search rather than on textual product names, a methodological point with direct consequences for the curation of phage genomes used in therapeutic candidate selection. Measured directly, that conservation must be qualified in one respect. Among endolysins from genomes of the same species, the catalytic domains are effectively fixed, the amidase domain being identical in every pair and CHAP differing by less than 1%, whereas the cell-wall-binding SH3b domain varies by up to 5.7%. Recognition-level variation is therefore not confined to the tail module: it is also present, at a smaller amplitude, in the binding domain of an otherwise invariant lytic enzyme. This is consistent with reports that SH3b-type binding domains of staphylococcal endolysins differ in strain-binding profile, and with the modular engineering of phage scaffolds in which binding specificity is exchanged independently of catalytic function,^(8)^ and it means that the conservation of the lysis module should be read as conservation of catalytic architecture rather than of the entire molecule. Between lineages the ranking reverses, CHAP being the most divergent domain, so no single domain can be described as the conserved or the variable one without stating the comparison scale.

The contrast between a conserved lysis module and an apparently variable host-recognition module does not survive re-annotation. Under domain- and structure-level annotation, host-recognition genes are detectable in every genome examined, and the variability visible in the deposited records is a property of those records. What the comparison does establish is that the two module classes differ in how reliably they are annotated: endolysin assignments were stable across all three sources, whereas holin and RBP assignments were not. This interpretation bears directly on the paradoxical biofilm behaviour of vB_SauM-515A1 reported previously:^(12)^ because its endolysin is intron-split rather than truncated, and the intact LysK retains the full catalytic architecture, its atypical biofilm response is unlikely to stem from endolysin deficiency; host recognition, translational compatibility, matrix-acting depolymerases and anti-defence systems remain equally plausible and as-yet-untested contributors. The two phages whose contrasting biofilm behaviour motivated this survey differ on a further axis that the present analysis makes measurable. vB_SauP-436A1 has the codon usage most similar to that of *S. aureus* in the dataset (r = 0.972) and encodes no tRNA, whereas vB_SauM-515A1 has the least similar (r = 0.844) and carries the three-tRNA macro-class complement; they also differ more than eight-fold in genome size and coding capacity. These are properties of the genome sequence, not measurements of biofilm activity, and no causal inference is drawn from them; they are noted because they identify translational compatibility, alongside host recognition, as a candidate variable that the original phenotypic comparison did not control and that would be testable experimentally. The paradox that motivated this survey is not confined to *Staphylococcus*. Phage predation has been reported to increase rather than reduce biofilm biomass across several bacterial genera, through mechanisms as different as lysis-driven release of extracellular DNA in *Shewanella oneidensis*,^(33)^ structural incorporation of filamentous phage into the *Pseudomonas aeruginosa* matrix,^(34)^ and coevolutionary selection for mucoid phenotypes in *Pseudomonas fluorescens*.^(35)^ A broader survey of biofilm formation under phage predation reaches the same conclusion.^(36)^ That the same paradoxical direction arises from unrelated mechanisms suggests it reflects a general property of phage-biofilm interaction rather than a peculiarity of any one host, and reinforces the central limitation of a genomic survey such as this one: sequence capacity does not predict the direction of the biofilm response. Whether the reconstituted enzyme is kinetically equivalent to a canonical LysK is not established. Splicing restores the CHAP-Amidase-SH3b architecture at the RNA level and the mature protein is reported as full length,^(28)^ so no structural basis for reduced activity is apparent; but neither specific activity nor turnover has been measured for the spliced product, and any residual difference would also depend on splicing efficiency in vivo, which is likewise unmeasured. The consequence for the present interpretation is the same either way. If the reconstituted enzyme is equivalent, the atypical biofilm response of vB_SauM-515A1 must be attributed to factors outside the lysis module; if it is not, the difference is a kinetic property of the enzyme rather than the absence or truncation of a module, and would still be invisible to a genomic survey. Distinguishing the two requires purified-enzyme kinetics on the spliced product, which lies outside the scope of a sequence-based analysis.

The phylogenetic analysis reinforces this conclusion. The near-zero internal branch lengths and consequent low internal support within the 17-phage group reflect the high conservation of TerL among these phages rather than a methodological limitation, since a single conserved marker such as TerL resolves inter-family relationships well but discriminates poorly among closely related intra-family members.^(21)^ Ninety-two of the 190 pairwise TerL distances were exactly zero, which is what that conservation amounts to in practice. The long branches and elevated GC content of EW and SA13 are mutually consistent and support independent evolutionary trajectories for these two phages. Importantly, the placement of vB_SauM-515A1 within the conserved core clade indicates that its atypical biofilm behaviour cannot be attributed to deep phylogenetic divergence. Nucleotide-level comparison refines this picture: Twort, although basal to the TerL 17-phage group, falls below the ICTV genus threshold against every other macro-class genome (34.5-36.8%), so its basal position on a single-marker tree reflects the conservation of the marker rather than membership of the same genus.

Among the accessory features catalogued here, phage-encoded tRNAs are of particular interest, and their distribution is informative only when the deposited annotation is set aside. De novo prediction recovered tRNA genes in 18 of 22 genomes rather than the five in which they had been annotated, and the four genomes without them are precisely the four whose codon usage most closely matches that of the host. Carriage of tRNAs and divergence from host codon usage therefore coincide completely in this dataset, with no genome falling between the two groups. The direction of that relationship cannot be established here — a phage whose codon usage has drifted from its host may be under selection to carry its own tRNAs, or carriage may relax the constraint that would otherwise keep its codon usage host-like — and the dataset contains no independent measure of translational efficiency with which to test either. Phage-encoded tRNAs have been proposed both as translational supplements that support phage protein synthesis when the host tRNA pool is suboptimal and, more recently, as anti-defence elements that counteract bacterial immune systems;^(37^ ^,38^ ^,39)^ the composition observed here does not discriminate between these roles, since the single host-rare codon decoded (GAC) is accompanied by decoding of the rest of its synonymous family. Both roles remain hypotheses requiring experimental confirmation in *S. aureus* phages and were not tested in the present, sequence-based analysis. More generally, the lysis and host-recognition cassettes are not the only modules that could contribute to differences in therapeutic response. The translational module examined here, comprising phage-encoded tRNAs and the codon usage of the phage genome, varies measurably across the dataset and separates it completely into two groups, and other modules not examined here, such as anti-defence systems and depolymerases acting on the biofilm matrix, are equally plausible contributors. The present survey characterises two cassettes because they are the two for which domain-level evidence can be obtained from sequence alone; it does not imply that they are the only relevant ones. This bears on what the survey can and cannot be used for. It does not license a selection strategy that simply seeks phages whose lysis and host-recognition modules most closely resemble the canonical ones, and the present results argue against such a strategy: the canonical architecture is so nearly universal in this dataset that it does not discriminate between candidates, and the two phages with the most divergent lysis modules are not the two with the divergent biofilm phenotypes. What a module-level survey does provide is the opposite service: it identifies which genomes carry a disrupted or non-canonical module and therefore require experimental verification before use, and it provides the domain-level inventory against which a phenotypic result can later be interpreted. Candidate selection remains a phenotypic decision informed by genomics, not replaced by it.

Several limitations should be acknowledged. The analysis is annotation-dependent: feature counts and module assignments derive from deposited GenBank records and computational domain inference, which approximate but do not replace experimental validation of protein function. The magnitude of that dependence is quantified in Table III: re-annotation changed the RBP call in eight of 22 genomes and the holin call in one, without any change to the underlying sequences. Detection also depends on the keyword vocabulary applied downstream of annotation, as the two micro-class genomes demonstrate; module surveys that report presence or absence without stating both the annotation source and the detection vocabulary are not comparable between studies. The study is descriptive and comparative; no statistical hypothesis testing was applied, and the modest size of the dataset limits generalisation. The phylogeny rests on a single conserved marker and therefore does not capture the mosaic, recombination-rich nature of phage genomes. Accordingly, the genomic features identified here are best regarded as hypothesis-generating signals that require subsequent experimental confirmation, in line with the role of this study as preliminary groundwork for planned experimental investigation. The codon usage comparison is descriptive and correlational: RSCU similarity and tRNA carriage covary completely in this dataset, but with 22 genomes falling into two size-stratified groups the association cannot be separated from lineage, and no causal or directional claim is made. The domain-partitioned identity analysis rests on the six unique endolysins in which all three domains were resolved, five of which come from genomes above the species demarcation threshold; the within-species figures therefore describe one lineage rather than the dataset as a whole. The phylogeny rests on a single conserved marker in genomes that are largely conspecific, so it supports statements about lineage separation but not about branching order within the 17-phage group; and the Twort tip is a partial sequence, as stated in Methods. No enzymatic measurement was made: where the manuscript reports an intact domain architecture it reports genomic capacity, not catalytic equivalence to a canonical enzyme.

*In conclusion* - This comparative genomic survey of lytic *S. aureus* bacteriophages shows that the lysis module is conserved at the level of domain architecture, and that this conservation is recovered identically from deposited and re-annotated records. Host-recognition genes appeared variable in the deposited annotation, being detectable in 14 of 22 genomes, but were recovered in 20 of 22 after re-annotation and in all 22 when the annotation sources were combined; their apparent variability therefore reflects annotation completeness rather than genomic content. Domain-level analysis distinguished true endolysins from virion-associated peptidoglycan hydrolases and revealed two independent mechanisms by which lysis-module genes can be interrupted — HNH endonuclease insertion and intron splitting — both of which can mislead annotation that relies on product names alone. The same effect extends beyond the lysis and host-recognition modules: de novo prediction recovered tRNA genes in 18 of 22 genomes against five in the deposited records, and the genomes lacking them proved to be those whose codon usage most closely matches the host. Module-level comparisons of phage genomes therefore require the annotation source to be stated, and re-derived wherever comparative claims are made. These findings provide a genomic foundation for the rational selection of phage candidates and a basis for experimentally investigating planktonic and biofilm-associated responses in subsequent work, in which *S. aureus*, as a major human pathogen, remains the clinically relevant target.

## SUPPLEMENTARY MATERIALS

Supplementary material

## Data Availability

All genome sequences analysed in this study are publicly available from the NCBI GenBank database under the accession numbers listed in Table I. The GenBank flat files (.gb) used as input, together with the custom Python analysis scripts that reproduce all reported results, are openly available in a public repository (https://github.com/HivanA98/In-silico-characterisation-of-lysis-and-host-recognition-modules-in-S.-aureus-bacteriophage-genomes), archived at a fixed version under release tag v3.2.3-MIOC-revision and deposited with the persistent identifier https://doi.org/10.5281/zenodo.21988477. No new nucleotide or protein sequences were generated in this work; therefore, no additional public-bank accession numbers were created. The re-annotated GenBank records produced by Pharokka, Phold and Phynteny, the three-arm detection outputs underlying Table III, and the codon usage matrices underlying Supplementary data (Table IV) are deposited in the same repository under a tagged release.
