# Supplementary material for: In silico characterisation of lysis and host-recognition modules in Staphylococcus aureus bacteriophage genomes
Source: Mem Inst Oswaldo Cruz. 2026 Nov 2;121:e260245. doi: 10.1590/0074-02760260245 (PMC13729910; doi:10.1590/0074-02760260245)
Supplement: Supplementary file 1 [file 1678-8060-mioc-121-e260245-s1.pdf]

Case-by-case description of non-canonical endolysin assignments

The four endolysin assignments that departed from the canonical LysK architecture, and the three in which automated keyword extraction returned a protein other than the free endolysin, are described individually below. Each case is summarised in Table II and its footnotes; the detail recorded here is how the assignment was reached.

Two mechanistically distinct departures from the canonical architecture were observed, both of which confound keyword-based annotation. In the Sb-1 lineage (Sb1M_6168, Sb1M_9832) the endolysin open reading frame is interrupted by an HNH endonuclease insertion, leaving no intact endolysin-sized frame in the deposited inventory: a 57-amino-acid LysM-domain protein and a 166-amino-acid putative HNH endonuclease were annotated separately within the lysis-module region, and a 141-amino-acid CHAP fragment was recovered by tBLASTn. In vB_SauM-515A1 and vB_SauM-fRuSau02 the *lysK* gene is interrupted by a self-splicing group I intron carrying the ksaI homing endonuclease; the CHAP domain lies on the opposite side of the intron, and the intact CHAP-Amidase-SH3b enzyme is reconstituted at the RNA level.

In three further genomes the automated keyword extractor returned a protein that was not the free endolysin. In Maine a non-lytic YbiA-like N-glycosidase was returned, and a canonical 495-amino-acid LysK was recovered by tBLASTn at 99% identity. In JD007 a 295-amino-acid virion-associated NlpC/P60 protein was returned, and a 495-amino-acid free LysK was recovered by tBLASTn at 99% identity. In Twort a 1269-amino-acid phage tail lysozyme (Phage_lysozyme2), tail-anchored and therefore a virion-associated peptidoglycan hydrolase rather than a free endolysin, was returned, and only a partial, divergent LysK region (45% identity over 456 amino acids) was recovered.

TABLE I

Intergenomic similarity of the 24 genomes analysed (22 retained plus two excluded during curation), computed with VIRIDIC using default BLASTN parameters. Values are percent intergenomic similarity; species and genus demarcation thresholds follow ICTV recommendations (95% and 70%, respectively)

genome	EW	SA13	vB_SauP-436A1	Portland	NC_055906_Staphylococcus_phage_vB_SauP_EBHT	MW248466_Staphylococcus_phage_MarsHill	Twort	qdsa002	VB_SavM_JYL01	Maine	JD007	Sb-1	A3R	vB_SauM-515A1	vB_SauM-fRuSau02	MSA6	676Z	Fi200W	P4W	A5W	Staph1N	Sb1_8383	Sb1M_6168	Sb1M_9832
EW (NC_007056)	100	27.45	0	0.424	0.424	0.094	0.52	0.423	0.359	0.359	0.36	0.39	0.359	0.347	0.347	0.348	0.347	0.345	0.347	0.353	0.352	0.364	0.367	0.367
SA13 (NC_021863)	27.45	100	0	0.269	0.269	0.117	0.326	0.136	0.13	0.171	0.175	0.147	0.092	0.131	0.131	0.131	0.115	0.131	0.115	0.133	0.133	0.137	0.138	0.138
vB_SauP-436A1 (MN150710)	0	0	100	87.91	87.91	0.183	0.311	0.264	0.315	0.255	0.221	0.265	0.238	0.245	0.245	0.245	0.244	0.231	0.25	0.231	0.231	0.259	0.261	0.261
Portland (MT926124)	0.424	0.269	87.91	100	100	0.183	0.178	0.212	0.241	0.322	0.212	0.196	0.156	0.188	0.188	0.188	0.189	0.233	0.211	0.193	0.192	0.199	0.2	0.2
NC_055906_Staphylococcus_phage_vB_SauP_EBHT	0.424	0.269	87.91	100	100	0.183	0.178	0.212	0.241	0.322	0.212	0.196	0.156	0.188	0.188	0.188	0.189	0.233	0.211	0.193	0.192	0.199	0.2	0.2
MW248466_Staphylococcus_phage_MarsHill	0	0.117	0.183	0.183	0.183	100	1.088	0.952	0.968	0.895	0.947	0.851	0.805	0.993	1.005	1	0.944	0.955	0.96	0.933	0.933	0.959	0.962	0.962
Twort (NC_007021)	0.52	0.326	0.311	0.178	0.178	1.088	100	34.839	35.399	34.915	35.175	36.841	35.393	34.88	34.617	34.697	34.786	34.876	34.792	34.545	34.54	35.474	35.657	35.655
qdsa002 (KY779849)	0.423	0.136	0.264	0.212	0.212	0.952	34.839	100	95.348	89.036	90.31	84.395	85.165	86.526	86.63	86.775	87.045	87.252	87.319	87.887	87.913	87.768	88.148	88.148
VB_SavM_JYL01 (MH159197)	0.359	0.13	0.315	0.241	0.241	0.968	35.399	95.348	100	86.195	87.289	81.983	82.559	83.617	83.736	83.873	84.161	84.356	84.656	85.314	85.341	85.069	85.433	85.433
Maine (MN045228)	0.359	0.171	0.255	0.322	0.322	0.895	34.915	89.036	86.195	100	93.537	87.967	87.537	89.402	89.159	89.337	90.159	89.865	89.572	90.143	90.182	90.808	91.209	91.209
JD007 (NC_019726)	0.36	0.175	0.221	0.212	0.212	0.947	35.175	90.31	87.289	93.537	100	87.063	87.899	88.98	89.097	89.276	89.818	89.927	90.086	90.704	90.744	90.601	90.949	90.949
Sb-1 (NC_023009)	0.39	0.147	0.265	0.196	0.196	0.851	36.841	84.395	81.983	87.967	87.063	100	92.235	93.488	93.111	93.117	92.532	92.804	92.495	93.206	93.173	95.367	95.349	95.349
A3R (NC_047723)	0.359	0.092	0.238	0.156	0.156	0.805	35.393	85.165	82.559	87.537	87.899	92.235	100	93.961	94.206	94.314	96.99	96.651	96.849	96.236	96.274	95.379	95.787	95.787
vB_SauM-515A1 (MN047438)	0.347	0.131	0.245	0.188	0.188	0.993	34.88	86.526	83.617	89.402	88.98	93.488	93.961	100	99.381	99.248	96.744	97.053	96.895	97.452	97.49	98.555	98.427	98.427
vB_SauM-fRuSau02 (MF398190)	0.347	0.131	0.245	0.188	0.188	1.005	34.617	86.63	83.736	89.159	89.097	93.111	94.206	99.381	100	99.823	96.965	97.277	97.117	97.737	97.713	98.538	98.555	98.555
MSA6 (NC_047726)	0.348	0.131	0.245	0.188	0.188	1	34.697	86.775	83.873	89.337	89.276	93.117	94.314	99.248	99.823	100	97.136	97.444	97.223	97.782	97.821	98.513	98.663	98.663
676Z (NC_047724)	0.347	0.115	0.244	0.189	0.189	0.944	34.786	87.045	84.161	90.159	89.818	92.532	96.99	96.744	96.965	97.136	100	99.376	99.094	98.2	98.238	98.146	98.557	98.557
Fi200W (NC_047725)	0.345	0.131	0.231	0.233	0.233	0.955	34.876	87.252	84.356	89.865	89.927	92.804	96.651	97.053	97.277	97.444	99.376	100	99.446	98.462	98.501	98.373	98.785	98.785
P4W (NC_047727)	0.347	0.115	0.25	0.211	0.211	0.96	34.792	87.319	84.656	89.572	90.086	92.495	96.849	96.895	97.117	97.223	99.094	99.446	100	98.924	98.962	98.193	98.605	98.605
A5W (EU418428)	0.353	0.133	0.231	0.193	0.193	0.933	34.545	87.887	85.314	90.143	90.704	93.206	96.236	97.452	97.737	97.782	98.2	98.462	98.924	100	99.961	98.797	99.216	99.216
Staph1N (NC_047722)	0.352	0.133	0.231	0.192	0.192	0.933	34.54	87.913	85.341	90.182	90.744	93.173	96.274	97.49	97.713	97.821	98.238	98.501	98.962	99.961	100	98.837	99.255	99.255
Sb1_8383 (MN336261)	0.364	0.137	0.259	0.199	0.199	0.959	35.474	87.768	85.069	90.808	90.601	95.367	95.379	98.555	98.538	98.513	98.146	98.373	98.193	98.797	98.837	100	99.7	99.7
Sb1M_6168 (MN336262)	0.367	0.138	0.261	0.2	0.2	0.962	35.657	88.148	85.433	91.209	90.949	95.349	95.787	98.427	98.555	98.663	98.557	98.785	98.605	99.216	99.255	99.7	100	99.999
Sb1M_9832 (MN336263)	0.367	0.138	0.261	0.2	0.2	0.962	35.655	88.148	85.433	91.209	90.949	95.349	95.787	98.427	98.555	98.663	98.557	98.785	98.605	99.216	99.255	99.7	99.999	100

VIRIDIC: virus intergenomic distance calculator; ICTV: International Committee on Taxonomy of Viruses.

CC BY

TABLE II

Pairwise percent amino-acid identity of endolysin domains, computed separately for each domain after alignment, restricted to the six unique endolysin sequences carrying all three domains. Within-species pairs are the ten pairs among five macro-class genomes; between-lineage pairs are the five pairs of SA13 against those five

Partition	Population	N_pairs	Min_pct	Max_pct
full_length	within-species (10 pairs, macro-class genomes)	10	98.4	100.0
full_length	between-lineage (5 pairs, SA13 vs macro-class)	5	42.7	43.0
CHAP	within-species (10 pairs, macro-class genomes)	10	99.2	100.0
CHAP	between-lineage (5 pairs, SA13 vs macro-class)	5	22.9	22.9
Amidase	within-species (10 pairs, macro-class genomes)	10	100.0	100.0
Amidase	between-lineage (5 pairs, SA13 vs macro-class)	5	54.2	54.5
SH3b	within-species (10 pairs, macro-class genomes)	10	94.3	100.0
SH3b	between-lineage (5 pairs, SA13 vs macro-class)	5	47.6	48.6

CHAP: cysteine, histidine-dependent amidohydrolase/peptidase; SH3b: bacterial Src-homology 3 domain.

TABLE III

Residual proteins of unknown function resolved by Phynteny for each genome, with PHROG-category assignment counts and per-genome median assignment confidence

Phage	Accession	CDS_total	Phold_unknown_function	Entering_Phynteny	Assigned_by_Phynteny	Assigned_to_tail	Median_confidence	Confidence_ge_0.8
676Z	NC_047724.1	274	155	152.0	152.0	0.0	0.221	3.0
A3R	NC_047723.1	252	143	140.0	140.0	0.0	0.227	3.0
A5W	EU418428.2	270	154	151.0	151.0	0.0	0.217	3.0
EW	NC_007056.1	82	32	32.0	32.0	0.0	0.266	0.0
Fi200W	NC_047725.1	268	151	146.0	146.0	0.0	0.219	5.0
JD007	NC_019726.1	247	138	136.0	136.0	0.0	0.223	3.0
MSA6	NC_047726.1	271	153	150.0	150.0	0.0	0.225	3.0
Maine	MN045228.1	245	138	134.0	134.0	0.0	0.248	5.0
P4W	NC_047727.1	269	150	147.0	147.0	0.0	0.219	4.0
Portland	MT926124.1	22	10	10.0	10.0	0.0	0.195	0.0
SA13	NC_021863.1	74	25	24.0	24.0	1.0	0.351	0.0
Sb-1	NC_023009.1	215	114	111.0	111.0	0.0	0.262	6.0
Sb1M_6168	MN336262.1	244	133	130.0	130.0	0.0	0.244	3.0
Sb1M_9832	MN336263.1	244	133	130.0	130.0	0.0	0.244	3.0
Sb1_8383	MN336261.1	251	140	137.0	137.0	0.0	0.24	4.0
Staph1N	NC_047722.1	270	154	151.0	151.0	0.0	0.216	3.0
Twort	NC_007021.1	214	119	117.0	117.0	0.0	0.247	8.0
VB_SavM_JYL01	MH159197.1	248	141	137.0	137.0	0.0	0.25	3.0
qdsa002	KY779849.1	256	148	145.0	145.0	0.0	0.236	2.0
vB_SauM-515A1	MN047438.1	276	158	155.0	155.0	0.0	0.224	2.0
vB_SauM-fRuSau02	MF398190.1	275	157	154.0	154.0	0.0	0.224	2.0
vB_SauP-436A1	MN150710.1	22	10	10.0	10.0	0.0	0.204	0.0

CDS: coding sequence; PHROG: prokaryotic virus remote homologous groups.

TABLE IV

Codon usage similarity between each phage genome and the pooled *Staphylococcus aureus* host reference, over the 59 codons of degenerate synonymous families

Accession	N_codons	GC3_phage_pct	GC3_host_pct	Pearson_r	Spearman_rho	Mean_abs_dRSCU
MN150710.1	5631	21.28	22.73	0.9722	0.9559	0.1805
MT926124.1	5551	21.31	22.73	0.97	0.9455	0.1712
NC_007056.1	14315	30.62	22.73	0.9493	0.9285	0.2083
NC_021863.1	13140	29.85	22.73	0.9265	0.9226	0.2188
NC_007021.1	39026	20.37	22.73	0.8808	0.7968	0.287
MH159197.1	42221	18.73	22.73	0.8539	0.8144	0.2886
KY779849.1	42829	19.18	22.73	0.8528	0.8086	0.2863
NC_019726.1	41684	18.98	22.73	0.8517	0.8174	0.2855
MN045228.1	42812	19.19	22.73	0.8495	0.8123	0.2886
NC_023009.1	36532	18.71	22.73	0.848	0.8072	0.2903
MN336263.1	35641	18.76	22.73	0.8478	0.8082	0.2904
MN336261.1	36565	18.74	22.73	0.8478	0.8079	0.2907
NC_047725.1	43881	19.45	22.73	0.8475	0.8079	0.2892
NC_047723.1	41696	19.42	22.73	0.8474	0.8101	0.2886
EU418428.2	43214	19.47	22.73	0.8471	0.8085	0.2882
MN336262.1	35865	18.7	22.73	0.847	0.8052	0.2905
NC_047722.1	43254	19.47	22.73	0.847	0.8085	0.2883
NC_047726.1	43777	19.28	22.73	0.8462	0.8123	0.2884
NC_047724.1	43904	19.42	22.73	0.8461	0.8084	0.2907
NC_047727.1	43606	19.43	22.73	0.8461	0.8077	0.2912
MF398190.1	43410	19.21	22.73	0.8457	0.8126	0.2888
MN047438.1	43526	19.17	22.73	0.8436	0.8115	0.29

GC3: guanine-plus-cytosine content at the third codon position; RSCU: relative synonymous codon usage; dRSCU: difference in RSCU.
