# Supplementary material for: In silico characterisation of lysis and host-recognition modules in Staphylococcus aureus bacteriophage genomes
Source: Mem Inst Oswaldo Cruz. 2026 Nov 2;121:e260245. doi: 10.1590/0074-02760260245 (PMC13729910; doi:10.1590/0074-02760260245)
Supplement: Peer review history

# Reviewer report: FIRST REVIEW ROUND - REVIEWERS’ COMMENTS

## REVIEWER #1

General Comments

Overall, the manuscript addresses an interesting topic by exploring the lysis and host-recognition modules in S. aureus phages to guide non-traditional therapeutic choices. While the study is primarily descriptive, the authors adequately acknowledge its limitations. My main concern relates to what is defined as “conserved” throughout the text, as this concept remains unclear and requires further specification.

Specific Points

The English language could be improved.

Lines 12-18 (Page 3): Has this type of paradox only been observed in Staphylococcus, or does it also occur in other species? A brief mention of this broader context would enrich the discussion.

Could only the lysis and host-recognition modules reflect differences in therapeutic response, or might other modules also play a role? Consequently, does this imply that the strategy for identifying clinically relevant phages relies on finding those with lysis and host-recognition modules similar to canonical ones? Please clarify this rationale.

The following reference is inaccessible: Abdraimova NK 2024 (doi: 10.24075/brsmu.2024.061). Please verify and provide a working link or correct the citation.

Is the codon usage of these phages similar to that of S. aureus? Phage replication is expected to be more efficient in a host with a similar codon usage profile. Discussing this could add depth to the host-phage dynamics.

In the Introduction, the lysis cassettes (holin and endolysin) are mentioned, but what constitutes the host-recognition cassette? Furthermore, since the study verifies the functional domains of these modules, it would be beneficial to briefly introduce these domains in the Introduction as well.

In Table II, it would be helpful to visually or categorically differentiate between the lysis and the host-recognition modules.

Lines 40-42 (Page 6) and Table II: What exactly does the “conservation” of these components refer to? Is it presence/absence, amino acid sequence length, or the presence of specific domains? It would be highly relevant to report the sequence identity of these sequences. Even if functional domains are conserved, sequence variations in other regions might indicate distinct selective pressures.

Lines 31-32 (Page 9): Again, does “intact” refer to the protein’s length or to 100% sequence identity? Please specify.

Lines 54-55 (Page 6): Please define the RBP genes. Additionally, could you briefly explain how the tail fiber determines the host range?

In the results, lysins are well described, detailing various cases where LysK is non-canonical. However, to improve the manuscript’s readability and dynamism, I would suggest moving a portion of these descriptive details to a Supplementary Material.

I expected a more specific comparative analysis of the phages vB_SauP-436A and vB_SauM-515A1, which were cited in the Introduction as inducing distinct biofilm biomasses. Elaborating on this comparison would strengthen the study’s narrative connection to the introduction.

Line 46 (Page 9): References 24 and 26 do not seem appropriate to support this statement.

Lines 47-48 (Page 9): Would an intron-split endolysin, once processed, have the same catalytic efficiency as a canonical endolysin? Could there be structural or kinetic differences influencing the biofilm response? If the efficiency is indeed the same, then the biofilm’s behavior must be attributed to other factors.

## REVIEWER #2

Well-curated dataset, explicit criteria, clear writing. The most valuable contribution is re-examining deposited annotations against curated domain databases instead of trusting product names. That result currently sits as an intermediate step, while the result given prominence is unsupported.

Major

1. The central comparison is not supported and the paper needs reframing around what it actually shows. No host-range data are analysed, yet the Objectives and Conclusion assert that host recognition governs host range and is the rational target for engineering. The asymmetry beneath this claim is the core problem: endolysins were characterised with InterPro, tBLASTn and manual curation, while holin and RBP were scored by keyword matching alone. Domain-level characterisation is being compared against text matching and the difference reported as biology. The 14/22 figure cannot mean what it is taken to mean — a tailed phage cannot lack a receptor-binding apparatus, and Table II shows the artefact directly, with Sb-1 scored positive while Sb1_8383, Sb1M_6168 and Sb1M_9832 are negative despite being treated as one lineage. Holin fails the same way. Both figures are lower bounds on annotation completeness, not measures of variability.

2. Re-annotate with Pharokka, Phold and Phynteny. The thesis is that deposited annotations are unreliable, yet the entire analysis rests on them. This is what makes comment 1 answerable rather than merely conceded.

3. “Highly conserved” and “markedly variable” carry no measurement. Supply pairwise identity across the canonical endolysins resolved by domain — if SH3b varies while the catalytic domains do not, recognition is not confined to the tail module and the central dichotomy collapses. The same imprecision governs a curation decision: one genome was excluded as “essentially identical” to another while two genomes of identical length were retained. State the threshold.

4. Reproducibility. Missing: keyword sets, tBLASTn parameters, MAFFT version, curation funnel, and a DOI with fixed commit. Deposit the TerL alignment.

Minor

1. Table II is titled as validated by InterPro, but InterPro was applied only to endolysins; the RBP column is a binary keyword call.

2. Figure 1 does not work as a visual. Beyond resolution, the tree is currently decorative: the manuscript narrates lineages, size classes, architecture exceptions and the two disruption mechanisms in text, none of which is visible on the tree. Consider rebuilding it in iTOL or FigTree and annotating the tips with those layers, such as family, genome size, endolysin architecture, HNH insertion and intron split, and the RBP call, so the figure carries the comparison rather than restating it.

3. The Ethics section is disproportionate for a computational study.

4. The AI-assistance declaration is misplaced. Its content is appropriate, but Acknowledgements is for human and institutional contributions that do not meet authorship criteria, and a tool cannot be acknowledged in that sense. Move it to a dedicated Declaration of AI Use section, or to Conflict of Interest if the journal has no such heading.

## AUTHORS’ RESPONSE TO THE REVIEWERS

Dear Dr Vicente,

We thank you and both reviewers for a review that improved this manuscript substantially. Reviewer 2 identified a claim that our data did not support, and correcting it required re-analysing the entire dataset under three annotation sources rather than one. That work changed the paper’s central result, and we are grateful for it. Reviewer 1’s insistence on defining “conserved” led us to measure what we had previously only asserted, with a result that partly contradicts our original framing.

Every comment is addressed below in the order received. Changes in the manuscript are highlighted. All code, genome records, alignments, intermediate results and the host profile are deposited at https://doi.org/10.5281/zenodo.21988477 under release tag v3.2.3-MIOC-revision.

Yours sincerely,

Ivan Armadi Hasugian, on behalf of both authors

Summary of principal changes

Reframing — No claim about host range remains. The 14/22 figure is reported as a lower bound on annotation completeness.

New Table III — Detection of holin and RBP across three annotation sources, all 22 genomes.

New Supplementary Table S1 — Residual annotation resolved by Phynteny.

New Supplementary Table S2 — Phage-versus-host codon usage with de novo tRNA counts.

New Supplementary Table S3 — VIRIDIC intergenomic similarity, 24 genomes.

New Supplementary Table S4 — Endolysin per-domain pairwise identity.

New Supplementary Text S1 — Case-by-case description of non-canonical endolysin assignments.

Figure 1 — Rebuilt in iTOL with five annotation layers; tree corrected after a fix to the terminase extractor; supplied as TIFF at 300 dpi.

Table II — New RBP InterPro domain column, corrected caption, two new footnotes, host-recognition columns shaded.

New Methods subsections — Intergenomic similarity; Domain-partitioned pairwise identity; Re-annotation and annotation-source comparison; Codon usage and phage-encoded tRNAs.

New references — 31-39, covering RSCU, tRNAscan-SE, Pharokka, Phold, VIRIDIC and four cross-genus biofilm studies.

Data availability — Zenodo DOI 10.5281/zenodo.21988477, release tag v3.2.3-MIOC-revision.

## Reviewer 2

We have placed Reviewer 2 first because the reframing requested in Major 1 determined the structure of the whole revision.

Major 1 — The central comparison is not supported and the paper needs reframing

Comment. No host-range data are analysed, yet the Objectives and Conclusion assert that host recognition governs host range. Endolysins were characterised with InterPro, tBLASTn and manual curation, while holin and RBP were scored by keyword matching alone. The 14/22 figure cannot mean what it is taken to mean.

Response. We accept this in full. The asymmetry was real and the claim was unsupported. We re-processed all 22 genomes through the identical S1-S4 detection funnel under three annotation sources - the deposited GenBank records, Pharokka v1.10.1 and Phold v1.3.0 (ProstT5 embeddings with Foldseek structural search) - so that the annotation layer was the only variable. Holin detection rose from 21/22 to 22/22 and tail fibre/RBP from 14/22 to 20/22 under both re-annotations, with RBP loci rising from 26 to 57 and 58. The four genomes the reviewer named behave exactly as predicted: Sb1_8383, Sb1M_6168 and Sb1M_9832 each move from zero to three RBP loci while Sb-1 remains at three, removing the within-lineage discordance. Taking the union of the three sources, holin and RBP are each detected in 22/22 genomes.

Response. The analysis requested in Major 3 supplied an independent confirmation that does not depend on re-annotation at all. Of the 231 genome pairs among the 22 retained genomes, 67 reach or exceed the ICTV species demarcation threshold of 95%, and 38 of those 67 are discordant in the deposited RBP call - the most extreme at 99.8% intergenomic similarity. Genome pairs that are 99-100% identical cannot differ in the presence of an entire gene family, so those discordances are necessarily annotation artefacts.

Response. We also report two genomes moving in the opposite direction. vB_SauP-436A1 and Portland are RBP-positive in the deposited annotation and negative after re-annotation, because our own keyword vocabulary does not capture the product names assigned to the short tail fibres of these micro-class genomes. We state this as a limitation of the detection layer rather than a biological finding.

Action. The Objectives, Results, Discussion and Conclusion were rewritten; no claim about host range remains anywhere in the manuscript. The figure of 14/22 is now reported as a lower bound on the completeness of deposited annotation. Table III (new) gives the three-arm comparison for all 22 genomes. A new Methods subsection, “Re-annotation and annotation-source comparison”, describes the procedure, and a new Results subsection reports it. The same effect proved to extend to a third feature class and is now also reported: de novo prediction recovers tRNA genes in 18 of 22 genomes against five in the deposited records.

Major 2 — Re-annotate with Pharokka, Phold and Phynteny

Comment. The thesis is that deposited annotations are unreliable, yet the entire analysis rests on them.

Response. All three tools were run. Pharokka and Phold are reported as full detection arms in Table III. Phynteny assigns a PHROG functional category to proteins that remain of unknown function rather than a product name, so it cannot generate module calls on the same basis as the other two; presenting it as a fourth column would imply an independent confirmation that it does not provide. To settle the point empirically rather than by argument, we nevertheless split the Phynteny output into one file per genome and re-processed all 22 through the identical funnel: the results match the Phold arm genome for genome on holin presence, on RBP presence and on RBP locus count. Phynteny assigned a category to all 2,599 proteins that remained of unknown function after Phold, but at low confidence (median 0.23; 2.5% at or above 0.8), and one protein in the entire dataset was assigned to the tail category.

Action. Pharokka and Phold results are in Table III and in the new Results subsection. The Phynteny result is reported in Supplementary Table S1, with the reasoning above stated in Methods. All four arms are deposited in the code archive.

Major 3 — “Highly conserved” and “markedly variable” carry no measurement

Comment. Supply pairwise identity across the canonical endolysins resolved by domain - if SH3b varies while the catalytic domains do not, recognition is not confined to the tail module. The same imprecision governs a curation decision: one genome was excluded as “essentially identical” to another while two genomes of identical length were retained. State the threshold.

Response. Both halves are now measured, and the first partly contradicts our original framing, which we report rather than soften. The unique endolysins were partitioned at their InterPro-resolved domain boundaries and each domain set aligned separately. Among the six sequences carrying all three domains, conservation proved to be scale-dependent with opposite rank order at the two scales. Within species the amidase domain is invariant at 100% across all ten pairs, CHAP ranges 99.2-100%, and SH3b ranges 94.3-100% - so SH3b is the only domain to fall below 95%, exactly as the reviewer anticipated. Between SA13 and those five sequences the order reverses: CHAP is the most divergent at 22.9%, SH3b intermediate at 47.6-48.6%, and amidase the least divergent at 54.2-54.5%. Recognition-level variation is therefore not confined to the tail module: it is present, at smaller amplitude, in the binding domain of an otherwise invariant lytic enzyme.

Response. For the curation threshold, we quantified intergenomic similarity with VIRIDIC using the default BLASTN parameter set and the ICTV demarcation thresholds. vB_SauP_EBHT is 100.0% similar to Portland, which is why it was excluded. However, applying the same criterion consistently shows that our original rule was not applied consistently: A5W/Staph1N and Sb1M_6168/Sb1M_9832 also reach 100.0% and were retained. Rather than defend an inconsistent rule we have changed the stated rule. The dataset is deliberately not dereplicated at the species level, because the object of study is the consistency of annotation rather than the extent of genomic diversity - and it is precisely the retention of near-identical genomes that makes the annotation discordance reported under Major 1 detectable.

Action. A new Methods subsection, “Domain-partitioned pairwise identity”, describes the procedure; the results are in the Results and in Supplementary Table S4. A new Methods subsection, “Intergenomic similarity”, states the VIRIDIC parameters and thresholds; the curation paragraph was rewritten with the numerical threshold, and the full matrix is Supplementary Table S3. “Conserved” is now defined operationally in the Results as pairwise amino-acid identity within a defined domain, not as the presence of a domain.

Major 4 — Reproducibility

Comment. Missing: keyword sets, tBLASTn parameters, MAFFT version, curation funnel, and a DOI with fixed commit. Deposit the TerL alignment.

Response. All five are now supplied. The keyword sets are in the deposited host profile: holin (5 terms), RBP (12), lysis (16, a deliberately broad collection net) and an 18-term precision denylist of broad-keyword false positives confirmed non-endolysin by InterPro. The tBLASTn command, its parameters and the 90% acceptance threshold are stated in Methods; we also note there that the outcome of each search is recorded in the profile as a curated case, so that re-running the pipeline reproduces the resolved assignment without repeating the search. MAFFT was the online service, run 16 August 2026 with the L-INS-i strategy and all other settings at default; the full MEGA 12.1.2 parameter set is given in Methods. The curation funnel is documented in the archive.

Action. The archive is deposited at https://doi.org/10.5281/zenodo.21988477 under release tag v3.2.3-MIOC-revision, which fixes the commit. It contains the code, the host profile, the deposited and re-annotated genome records, the InterPro output, the VIRIDIC matrix, the per-stage results for every arm, and the alignments - including the TerL alignment and the four endolysin domain alignments. The Data Availability statement cites the DOI and the tag.

Minor 1 — Table II is titled as validated by InterPro

Comment. InterPro was applied only to endolysins; the RBP column is a binary keyword call.

Response. Correct. In the revision InterPro was additionally applied to the RBP candidates in all three annotation arms. Domain support was recovered for 11 of 22 genomes in each arm, although not for the same 11: eight were supported in all three and the union across arms was 14 of 22. This is lower than the keyword-based call in every arm, and we report it as such rather than as an improvement: receptor-binding proteins are sparsely represented in profile-HMM databases, which is why structural homology as implemented in Phold, rather than domain assignment, is the appropriate symmetric comparator for this module class.

Action. The caption of Table II was corrected to state which validation applies to which column. A new column, RBP InterPro domain, was added, and two footnotes now state the evidence type behind each call. The three host-recognition columns are shaded to separate them visually from the lysis columns, which also addresses a request from Reviewer 1. We also note, for reproducibility, that the archive contains two versions of the endolysin extraction script: S4_endolysin_extractor_for_interpro.py is the standalone script used for the original submission and S4_endolysin_extractor.py the profile-driven version used throughout the revision. Both are deposited so either version of Table II can be reproduced; the revision used the latter throughout.

Minor 2 — Figure 1 does not work as a visual

Comment. The tree is currently decorative. Consider rebuilding it in iTOL or FigTree and annotating the tips with those layers, so the figure carries the comparison rather than restating it.

Response. Figure 1 was rebuilt and now carries five tip-level layers drawn from Tables I, II and III: family, genome size, endolysin domain architecture, lysis-gene disruption (HNH insertion and intron split), and the receptor-binding protein call before and after re-annotation. The last layer makes the central result of the revision visible directly: eight genomes show an empty square for the deposited call beside a filled square for the re-annotated call, and all eight lie inside the group whose members are at or above the species demarcation threshold to one another.

Response. Rebuilding the figure also required correcting the tree itself. The terminase extractor now returns the longest terminase-annotated coding sequence rather than the first, which changes the sequence used for Twort, whose terminase is split across three coding sequences by HNH insertions. Branch lengths, node support and the rooting were updated accordingly. We also state in Methods that the Twort tip is a 286-amino-acid fragment for which InterPro returns no domain, and that its separation from the remaining genomes is independently supported by intergenomic similarity (34.5-36.8%, below the ICTV genus threshold).

Action. Figure 1 was rebuilt in iTOL, is supplied as TIFF at 300 dpi, and the legend now states the annotation layers, the bootstrap support range for nodes inside the 17-phage group (27-64%), and the partial status of the Twort sequence. The iTOL annotation files are deposited with the tree.

Minor 3 — The Ethics section is disproportionate

Comment. The Ethics section is disproportionate for a computational study.

Response. Agreed. We have reduced it to a single sentence rather than removing it altogether, so that the absence of an ethics requirement is still stated explicitly for a study that uses only public sequence data.

Action. The Ethics paragraph in Materials and Methods was reduced from a full paragraph to one sentence.

Minor 4 — The AI-assistance declaration is misplaced

Comment. Acknowledgements is for human and institutional contributions; a tool cannot be acknowledged in that sense. Move it to a dedicated Declaration of AI Use section.

Response. Agreed.

Action. The declaration was moved to a dedicated Declaration of AI Use section, and the Acknowledgements now contain only institutional acknowledgement.

## Reviewer 1

General comment — the definition of “conserved”

Comment. My main concern relates to what is defined as “conserved” throughout the text, as this concept remains unclear and requires further specification.

Response. This was the most consequential comment we received from either reviewer, and answering it changed a claim rather than only a wording. Conservation is now defined operationally and measured. The full response is given under Reviewer 2, Major 3; in summary, conservation is reported as pairwise amino-acid identity within a defined domain, not as the presence of a domain, and it proved to be scale-dependent with opposite rank order within and between lineages.

Action. A definition sentence was added to the Results; the measurement is reported there and in Supplementary Table S4; a new Methods subsection describes the procedure.

- The English language could be improved

Response. The manuscript has been revised throughout for language. Overlong sentences in the Introduction, Results and Discussion were divided, redundant constructions removed, and spelling and terminology made consistent (British spelling throughout; “tail fibre” and “behaviour” standardised).

Action. Language revisions are distributed throughout and are highlighted with all other changes.

- Has this type of paradox only been observed in Staphylococcus?

Comment. Has this type of paradox only been observed in Staphylococcus, or does it also occur in other species? A brief mention of this broader context would enrich the discussion.

Response. It is not confined to Staphylococcus, and the mechanisms differ between the reported cases, which is itself informative. Phage predation has been reported to increase rather than reduce biofilm biomass through lysis-driven release of extracellular DNA in Shewanella oneidensis, structural incorporation of filamentous phage into the Pseudomonas aeruginosa matrix, and coevolutionary selection for mucoid phenotypes in Pseudomonas fluorescens. That the same paradoxical direction arises from unrelated mechanisms suggests a general property of phage-biofilm interaction rather than a peculiarity of one host.

Action. Two sentences and four references were added to the Discussion, and used to reinforce the limitation that sequence capacity does not predict the direction of the biofilm response. We kept this brief, as the reviewer asked for a mention of broader context rather than a review.

- Might other modules also play a role, and does this imply a selection strategy?

Comment. Could only the lysis and host-recognition modules reflect differences in therapeutic response, or might other modules also play a role? Consequently, does this imply that the strategy for identifying clinically relevant phages relies on finding those with lysis and host-recognition modules similar to canonical ones? Please clarify this rationale.

Response. Other modules certainly may. The translational module examined here - phage-encoded tRNAs and the codon usage of the phage genome - varies measurably across the dataset and separates it completely into two groups, and modules we did not examine, such as anti-defence systems and depolymerases acting on the biofilm matrix, are equally plausible contributors. We characterised these two cassettes because they are the two for which domain-level evidence can be obtained from sequence alone, not because they are the only relevant ones.

Response. On the second half of the question, our results argue against such a selection strategy. The canonical architecture is so nearly universal in this dataset that it does not discriminate between candidates, and the two phages with the most divergent lysis modules are not the two with the divergent biofilm phenotypes. What a module-level survey provides is the opposite service: it identifies which genomes carry a disrupted or non-canonical module and therefore require experimental verification before use, and it supplies the domain-level inventory against which a later phenotypic result can be interpreted. Candidate selection remains a phenotypic decision informed by genomics, not replaced by it.

Action. Both points were added to the Discussion.

- The Abdraimova NK 2024 reference is inaccessible

Comment. Please verify and provide a working link or correct the citation.

Response. We verified the citation, which is correct: Bull Russ State Med Univ 2024, issue 6, pages 98-104. The DOI resolves in a browser but is refused to automated agents, which is the likely source of the difficulty.

Action. The journal archive URL was added alongside the DOI in the reference list.

- Is the codon usage of these phages similar to that of S. aureus?

Comment. Phage replication is expected to be more efficient in a host with a similar codon usage profile. Discussing this could add depth to the host-phage dynamics.

Response. This was a substantive omission and the analysis produced a result we had not anticipated. Relative synonymous codon usage was computed for each phage genome and compared with the host over the 59 codons of degenerate synonymous families. Codon usage is broadly host-like across the dataset (Pearson r 0.844-0.972), but the dataset separates completely into two groups with no intermediate value: the four genomes in which de novo prediction detects no tRNA are the four most similar to the host (r = 0.927-0.972), while the 18 tRNA-carrying genomes are the least similar (r = 0.844-0.881). The ordering is not explained by base composition - EW and SA13 depart from the host GC3 by more than seven percentage points yet resemble the host more closely in codon preference than the macro-class genomes, whose GC3 lies within four points of the host. We are explicit that the relationship is correlational and that its direction cannot be determined from sequence data.

Action. A new Methods subsection, “Codon usage and phage-encoded tRNAs”, and a new Results subsection were added; the Discussion paragraph on phage-encoded tRNAs was rewritten around the result; the per-genome data are in Supplementary Table S2.

- What constitutes the host-recognition cassette, and its domains, in the Introduction

Comment. In the Introduction, the lysis cassettes are mentioned, but what constitutes the host-recognition cassette? It would be beneficial to briefly introduce these domains in the Introduction as well.

Response. Agreed. The Introduction previously described the lysis cassette without its counterpart.

Action. A passage was added to the Introduction defining receptor-binding proteins as the tail fibres, tailspikes and baseplate-associated proteins that make first contact with the bacterial surface, noting that in Staphylococcus these recognise wall teichoic acid and its glycosylation pattern, and describing the modular N-terminal anchoring and C-terminal specificity architecture. This also answers the reviewer’s later request to define the RBP genes and to explain how the tail fibre determines host range.

- Differentiate the lysis and host-recognition modules in Table II

Comment. In Table II, it would be helpful to visually or categorically differentiate between the lysis and the host-recognition modules.

Action. The three host-recognition columns of Table II are now shaded, separating them from the lysis columns.

- Does “intact” refer to length or to 100% identity?

Comment. Lines 31-32 (Page 9): does “intact” refer to the protein’s length or to 100% sequence identity? Please specify.

Response. Neither: it refers to the completeness of the domain architecture in the mature protein.

Action. A definition sentence was added to Methods: an endolysin is described as intact when its catalytic and cell-wall-binding domain architecture is complete in the mature protein, whether or not that protein is encoded by a single uninterrupted open reading frame; the term refers to domain architecture and not to sequence identity, which is reported separately in Supplementary Table S4.

Move a portion of the descriptive lysin detail to Supplementary Material

Comment. To improve the manuscript’s readability and dynamism, I would suggest

- moving a portion of these descriptive details to a Supplementary Material.

Comment. To improve the manuscript’s readability and dynamism, I would suggest moving a portion of these descriptive details to a Supplementary Material.

Action. The two case-by-case Results paragraphs were condensed into one and the detail moved to Supplementary Text S1. All seven cases remain named in the main text with their mechanisms; only the per-case amino-acid evidence was moved. The Results section is shorter by 278 words with no loss of finding.

- A more specific comparison of vB_SauP-436A and vB_SauM-515A1

Comment. Elaborating on this comparison would strengthen the study’s narrative connection to the introduction.

Response. The two phages sit at opposite extremes of the codon usage distribution: vB_SauP-436A1 is the most host-like genome in the dataset (r = 0.972) and encodes no tRNA, whereas vB_SauM-515A1 is the least host-like (r = 0.844) and carries the three-tRNA macro-class complement. They also differ more than eight-fold in coding capacity and in endolysin architecture, the latter being intron-split in vB_SauM-515A1. We present these as genomic axes on which the two differ, not as an explanation of the biofilm phenotype, which a sequence-based analysis cannot supply.

Action. The comparison was added to the Discussion and the corresponding conclusion retained in the Abstract.

- References 24 and 26 do not seem appropriate

Comment. Line 46 (Page 9): References 24 and 26 do not seem appropriate to support this statement.

Response. Agreed on both counts. Neither reference supported the sentence as written.

Action. The citation was removed from that sentence. Reference 26 is now cited in the Introduction, where it supports the account of host-range redirection through tail-fibre modification, and reference 30 was relocated to the Discussion sentence on modular exchange of binding specificity. Neither reference is orphaned.

- Would an intron-split endolysin have the same catalytic efficiency?

Comment. Could there be structural or kinetic differences influencing the biofilm response? If the efficiency is indeed the same, then the biofilm’s behaviour must be attributed to other factors.

Response. We cannot settle this, and we say so. Splicing restores the CHAP-Amidase-SH3b architecture at the RNA level and the mature protein is reported as full length, so no structural basis for reduced activity is apparent; but neither specific activity nor turnover has been measured for the spliced product, and any residual difference would also depend on splicing efficiency in vivo, which is likewise unmeasured. The consequence for our interpretation is the same either way, as the reviewer notes: if the enzyme is equivalent, the atypical biofilm response of vB_SauM-515A1 must be attributed to factors outside the lysis module; if it is not, the difference is a kinetic property of the enzyme rather than the absence or truncation of a module, and would still be invisible to a genomic survey.

Action. The passage above was added to the Discussion, together with a sentence in the limitations stating that where the manuscript reports an intact domain architecture it reports genomic capacity and not catalytic equivalence. We name the experiment that would settle it: purified-enzyme kinetics on the spliced product.

# Reviewer report: REVIEWERS’ COMMENTS

## REVIEWER #1

Here are a few minor considerations I still have:

-Species names should be italicized throughout the text.

-Did the codon usage analysis use only one S. aureus genome as a reference? I understand that the genome of the phage host strain is not always available, but its use would allow for a more accurate analysis.

-The title of Table II contains explanatory text that should be moved to footnotes.

-The citation order of the Supplementary Tables appears to be incorrect. For instance, ST1 is cited on page 22 of the proof, whereas ST3 and ST4 are first cited on pages 17 and 19, respectively.

-On page 26 of the proof, the sentence “InterPro domain support was recovered for the RBP candidates of 11 of 22 genomes” is repeated twice in a row.

-In the conclusions, the statement “Endolysin deficiency does not explain the biofilm paradox of vB_SauM-515A1” appears without prior context, making it seem disconnected.

-Finally, I still find the manuscript overly dense and detailed. The main text could be more concise, with finer details moved to the supplementary material. Furthermore, when tables are cited, the text exhaustively describes their data rather than simply highlighting the most relevant findings. While I believe the writing could still be streamlined, I do not consider this an impediment to accepting the work.

## REVIEWER #2

Thank you for the revision. You’ve correctly addressed all four major comments.

Objectives/Results/Discussion/Conclusion are consistent throughout. No further round needed from me.

One minor fix before production: a paragraph in Results (RBP InterPro support, 11/22 per arm) is duplicated almost verbatim.

Manuscript is ready from my side once that’s fixed.

## AUTHORS’ RESPONSE TO THE REVIEWERS

Dear Dr Vicente,

We thank both reviewers for a careful second reading. Every point raised has been addressed. One of them — the duplicated sentence in the Results, which both reviewers identified independently — was an editing error on our part that survived the first revision, and we are grateful it was caught before production.

All changes in this revision are highlighted in the manuscript file. Highlighting from the first revision was removed, so everything marked in the present version is new to this round.

Yours sincerely,

Ivan Armadi Hasugian, on behalf of both authors

Summary of changes in this revision – 2

Species names — 99 occurrences italicised across text, tables, footnotes, figure legend and supplementary captions.

Methods, codon usage — Now names the five pooled S. aureus reference genomes instead of referring to a single reference genome.

Table II caption — Reduced to a title; explanatory sentence moved to new footnote g.

Supplementary tables — Renumbered by order of first citation (VIRIDIC to S1, domain identity to S2, Phynteny to S3, codon usage to S4); caption blocks physically reordered; all nine in-text citations updated.

Results — Duplicated sentence and duplicated trailing clause removed from the annotation-source subsection.

Abstract — Orphan sentence on vB_SauM-515A1 removed from MAIN CONCLUSIONS; abstract now 190 words.

Unchanged — No figure, table value, reference or scientific claim was altered in this revision.

## Reviewer 2

We address Reviewer 2 first because the single point raised is the same one Reviewer 1 also identified.

Duplicated paragraph in Results

Comment. One minor fix before production: a paragraph in Results (RBP InterPro support, 11/22 per arm) is duplicated almost verbatim.

Response. Confirmed and corrected. The defect was narrower than a whole paragraph but had the same effect: in the annotation-source subsection, the sentence beginning “InterPro domain support was recovered for the RBP candidates of 11 of 22 genomes” appeared twice consecutively, and the clause that followed it — “lower than the keyword-based call in every case, consistent with the sparse representation of receptor-binding protein folds in profile-HMM databases...” — was also repeated. Both duplications arose when two versions of the sentence were merged during the first revision.

Action. The sentence and the trailing clause now appear once. No figure, number or claim changed; only the repetition was removed. Every reported value in that passage (11 of 22 per arm, eight supported in all three arms, union 14 of 22) is unaltered.

We also note Reviewer 2’s confirmation that the Objectives, Results, Discussion and Conclusion are now consistent, and that no further round is required from their side. We have made no other change to the substance of the manuscript in this revision.

## Reviewer 1

1. Species names should be italicised throughout the text

Comment. Species names should be italicized throughout the text.

Response. Corrected throughout. Italicisation was applied to every occurrence of the genus and species names in the running text, table cells, table footnotes, figure legend and supplementary captions, covering Staphylococcus aureus and its abbreviated form, Staphylococcus in “Staphylococcus phage”, and the three genera cited in the cross-genus passage of the Discussion (Shewanella oneidensis, Pseudomonas aeruginosa, Pseudomonas fluorescens).

Action. Ninety-nine occurrences were italicised. Genome accession identifiers and phage strain names were deliberately left in roman type, as these are not taxonomic names.

2. Did the codon usage analysis use only one S. aureus genome as a reference?

Comment. Did the codon usage analysis use only one S. aureus genome as a reference? I understand that the genome of the phage host strain is not always available, but its use would allow for a more accurate analysis.

Response. It did not, and we are grateful for the question because it exposed an inaccuracy in our Methods rather than in the analysis. The host reference codon table was computed by pooling the coding sequences of five curated S. aureus reference genomes — NCTC 8325 (NC_007795.1), Mu50 (NC_002758.2), MRSA252 (NC_002952.2), USA300_FPR3757 (NC_007793.1) and CP023390.1 — not from a single strain. Our Methods text referred to “the S. aureus reference genome” in the singular, which understated what was done and reasonably led to the reviewer’s question.

Response. We pooled deliberately rather than using one genome, for the reason the reviewer identifies: the propagating strain is not reported for most of these phage deposits, so no single strain can be claimed as the correct reference. Pooling across five strains that span the diversity of the species (methicillin-sensitive and methicillin-resistant, hospital- and community-associated lineages) gives a species-level codon table rather than a strain-level one, which is the level at which our comparison is made. We agree that using each phage’s own propagating host would be more accurate where that strain is known and sequenced, and this is stated as a limitation.

Action. The Methods sentence now names the five reference genomes and states explicitly that they were pooled rather than used singly.

3. The title of Table II contains explanatory text that should be moved to footnotes

Comment. The title of Table II contains explanatory text that should be moved to footnotes.

Response. Agreed. The caption carried a second sentence explaining which column rested on which kind of evidence. That sentence also duplicated material already present in footnotes e and f, so moving it removes a redundancy as well as shortening the title.

Action. The Table II caption is now a title only: “Domain architecture of lysis modules and presence of host-recognition modules among the 22 bacteriophages.” The explanatory sentence has been moved to a new footnote g.

4. The citation order of the Supplementary Tables is incorrect

Comment. The citation order of the Supplementary Tables appears to be incorrect. For instance, ST1 is cited on page 22 of the proof, whereas ST3 and ST4 are first cited on pages 17 and 19, respectively.

Response. Correct, and the problem was systematic rather than confined to those two tables. The four supplementary tables were numbered in the order in which they were written during the first revision, not in the order in which the text reaches them. First citation ran S3, S4, S1, S2.

Response. We renumbered by order of first citation rather than moving passages, because the positions of the citing passages are themselves determined by the argument: the intergenomic-similarity matrix is first needed in the dataset-curation paragraph of the Methods, and the domain-identity matrix in the paragraph defining “intact”. Reordering the text to fit the old numbering would have damaged the argument to preserve an arbitrary label.

Action. The supplementary tables were renumbered as follows, and the caption blocks were physically reordered to match: intergenomic similarity (VIRIDIC) is now Supplementary Table S1; endolysin per-domain pairwise identity is now S2; Phynteny residual annotation is now S3; codon usage similarity is now S4. All nine in-text citations were updated. First citation now runs S1, S2, S3, S4 in order. Supplementary Text S1 is a separate series and is unchanged.

5. A sentence in the Results is repeated twice in a row

Comment. On page 26 of the proof, the sentence “InterPro domain support was recovered for the RBP candidates of 11 of 22 genomes” is repeated twice in a row.

Response. Confirmed and corrected. This is the same defect Reviewer 2 identified; our response is given in full above, under Reviewer 2.

Action. The repetition and an associated repeated clause were removed. No reported value changed.

6. The Conclusions sentence on vB_SauM-515A1 appears without prior context

Comment. In the conclusions, the statement “Endolysin deficiency does not explain the biofilm paradox of vB_SauM-515A1” appears without prior context, making it seem disconnected.

Response. Agreed. The sentence sat in the MAIN CONCLUSIONS section of the structured abstract, where neither vB_SauM-515A1 nor the biofilm paradox had been introduced — the abstract reaches that sentence with no antecedent. It does not appear in the Conclusion section of the main text.

Response. We considered adding the missing context, but the abstract was already at the 200-word limit, so context could only be bought by removing a primary finding. The statement is a secondary result that is fully developed with its context in the Discussion, where the comparison between vB_SauP-436A1 and vB_SauM-515A1 is set out. We therefore removed it from the abstract rather than compress a primary finding to accommodate it.

Action. The sentence was deleted from MAIN CONCLUSIONS. The abstract is now 190 words. The finding itself is unchanged and remains in the Discussion with its full context.

7. The manuscript remains dense; details could move to supplementary material

Comment. Finally, I still find the manuscript overly dense and detailed. The main text could be more concise, with finer details moved to the supplementary material. Furthermore, when tables are cited, the text exhaustively describes their data rather than simply highlighting the most relevant findings. While I believe the writing could still be streamlined, I do not consider this an impediment to accepting the work.

Response. We accept the criticism and have not made further cuts in this revision, for a reason we would like to state rather than leave implicit. In the first revision we moved two case-by-case Results paragraphs to Supplementary Text S1, reducing that section by 278 words at the reviewer’s earlier request. The remaining density is concentrated in passages that exist because of the first round: the three-arm comparison, the domain-partitioned identity result and the intergenomic-similarity analysis were each added in direct response to specific comments, and each carries the numbers that support a claim now central to the paper.

Response. Cutting further would mean removing either those numbers or the qualifications attached to them, and our concern is that a shorter text would reintroduce exactly the kind of unsupported general statement that the first review asked us to eliminate. Given that the reviewer does not consider this an impediment to acceptance, we have preferred to leave the evidence in place. We would of course act on specific passages if the editor identifies any as expendable.

Action. No change. We note the point and would welcome specific guidance at the proof stage if further condensation is wanted.

# Reviewer report: REVIEWERS’ COMMENTS

## REVIEWER #1

All my questions were addressed.

## REVIEWER #2

No other comments.
